# Stoichiometric analysis of microbial communities links function, structure, and biomass carrying capacity

**DOI:** 10.1093/ismejo/wrag133

**Published:** 2026-07-03

**Authors:** Frank J Bruggeman, Timothy Paez-Watson, Bas Teusink, Robbert Kleerebezem

**Affiliations:** Systems Biology Lab, A-Life, AIMMS, VU University, De Boelelaan 1087, 1081 HV Amsterdam, The Netherlands; Systems Biology Lab, A-Life, AIMMS, VU University, De Boelelaan 1087, 1081 HV Amsterdam, The Netherlands; Laboratory of Systems and Synthetic Biology, Wageningen University and Research Centre, Stippeneng 4, 6708 WE Wageningen, The Netherlands; Systems Biology Lab, A-Life, AIMMS, VU University, De Boelelaan 1087, 1081 HV Amsterdam, The Netherlands; Environmental Biotechnology, TU Delft, Van der Maarsweg 9, 2629 HZ Delft, The Netherlands

**Keywords:** microbial ecology, ecological stoichiometric theory, principles of ecosystem assembly, metabolic flux analysis, catabolic product formation

## Abstract

Microbial communities carry out important ecological functions. Their activities emerge from interactions between species, often potentiated by metabolic traits. We lack a quantitative understanding of how these traits shape community properties. Here, we present theory for microbial communities, leveraging concepts from quantitative microbial physiology. We focus on how steady-state metabolic exchanges between species determine their fractional abundances, given their biomass and byproduct yields on nutrients. We start by deriving formal conditions for the steady states of communities of microbes that grow, die, and cross-feed metabolites. We describe the metabolic stoichiometry of nutrient uptake and the formation of biomass and byproducts for each species in terms of charge- and chemical-element balanced reactions (macrochemical reactions). Byproducts function as nutrients for other species. Next, we express the relative abundances of species (living and dead), the net metabolic conversion of a community, and the biomass carrying capacity in terms of the metabolic stoichiometry, growth rates and death rates of the species. We show how niche creation can emerge from stoichiometric imbalances in cross-feeding communities. Finally, we discuss how relative species abundances depend on the Adenosine Triphosphate (ATP) stoichiometries of intracellular metabolism.

## Introduction

Microbial communities contribute to cycling of chemical elements, human health, and biotechnology [1]. To forecast their responses to environmental changes (e.g. resilience and fragility) and to control their functions in applications, we need to understand them better. This is in particular relevant now, as many communities are under threat of climate change and pollution, and humanity needs to shift to more bio-based production systems [2].

Metagenomics revolutionized our insight into the genetic potential of the species making up communities [3, 4]. It remains challenging, however, to predict from this genomic knowledge emergent ecological mechanisms and microbial activities [5, 6]. This prevents us from rationally steering the behaviour of communities.

Microbial physiology has made great progress in our predictive understanding of the physiology of single microbes. This was primarily achieved by integrating theory, simulations, and quantitative data acquisition about, in particular, metabolic fluxes, and protein concentrations [7–12]. For instance, we have currently an understanding of why and when the model microbes *Escherichia coli, Saccharomyces cerevisiae*, and *Synechocystis* sp. switch in metabolic strategies in general terms such as optimal resource allocation, growth-rate maximization, and ATP yield [8, 9, 12–14]. We expect that these methods could also be adapted to microbiomes and thereby turned into powerful predictors of the behaviours of communities [15]. This would effectively turn microbial ecology from a descriptive field into a more predictive discipline, focusing on control, and steering of systems [2].

Here we focus on the translation of yield predictions from stoichiometric and thermodynamic considerations, originally developed for studies of single microbes [16–18], into a microbial ecology setting. Our methods allow for the prediction and understanding of the relative abundances of species and the net metabolic conversion and biomass carrying-capacity of the community, starting from the stoichiometric description of the metabolic activity and death rates of each of the species in the community. We limit this paper to the prediction of these systemic properties of the community at steady states. Steady states provide a natural starting point for understanding also more complex scenarios involving dynamics, because (stable) steady states serve as attractors in dynamic ecosystems [19]. In a follow-up study, we will focus on the dynamic description of microbial communities and these systemic quantities.

A central concept in this work is the macrochemical reaction which has a long history in microbial physiology and ecology [16, 17, 20]. One can think of the macrochemical reaction as the (chemical-element balanced, whole-cell) reaction converting substrates into biomass and byproducts, with a microbial species as the catalyst. A macrochemical reaction can be considered as a metabolic strategy of a microbe (e.g. it can grow via glucose respiration yielding carbon dioxide and water or via glucose fermentation giving rise to fermentation products). Microbes may shift between metabolic strategies when conditions change, affecting their product formation, cross-feeding interactions, and downstream metabolisms of other microbes. Metabolic interactions therefore set the relative abundances of microbes in addition to their death processes. The entire set of macrochemical reactions that a microbe can manifest is a measure of its metabolic plasticity; nowadays, it can be computed from the microbe’s genome through metabolic network reconstruction methods [21]. Macrochemical reactions can also be approximated from bioenergetic considerations, and an overview of their derivations and applications in industrial settings is provided elsewhere [17].

Here we use macrochemical reactions of microorganisms to predict stoichiometric properties of the community they compose, and subsequently derive the macrochemical reaction of the entire community (the community conversion) and determine the amount of biomass per unit energy source (the biomass carrying capacity).

## Materials and methods

All the figures were made in Wolfram Mathematica (version 14.3) and Biorender. All the mathematical derivations were checked in Wolfram Mathematica (version 14.3).

## Results

### Macrochemical-equation-based stoichiometric models of microbial communities

Macrochemical reactions (Table 1) result from the protein expression strategy of the species under the prevailing conditions and the assumption of a steady-state metabolism (balanced growth). Because during steady-state metabolism all intracellular metabolite concentrations remain constant, the chemical element composition, number of electrons, and charges of the growth substrates must balance with the composition of the growth products, including biomass. This elemental-conservation is captured in the macrochemical reaction, similarly as in any chemical reaction. The macrochemical equation can be calculated for any steady-state metabolic network from its stoichiometric matrix and steady-state flux distribution (see Appendix). Alternatively, a macrochemical equation can be inferred from experiments [16], estimated from chemical-element conservation and thermodynamic relations [17, 22], and from genome-scale stoichiometric model of metabolism [21].

**Table 1:** Explainer box with concise definitions for quick reference.

**Box — Key definitions**
**Macrochemical equation or macrochemical reaction**
A single, chemical-element balanced ‘overall’ reaction that summarizes which substrates are consumed and products (including biomass) are produced at a given physiological state. The stoichiometric coefficient that occur in a macrochemical equation are yields.
**Steady-state metabolism and balanced growth**
A cellular state during which the intracellular concentrations, enzyme activities, and the growth rate remains constant in time. This is the state of exponential growth achieved in batch and chemostat cultures.
**Yield**
The amount of product made per unit substrate. For instance, mol ATP per mol energy source (e.g. glucose) or per mol of oxygen consumed, gram biomass per mol carbon source or mol ethanol per mol carbon source.
**Community conversion**
The community’s macrochemical equation obtained by combining macrochemical equations of the member species, according to their relative contributions (weights).
**Biomass carrying capacity**
The maximum biomass that can be generated from a finite supply of substrates, based purely on stoichiometric constraints from the (community) macrochemical equation.
**Metabolic plasticity**
The ability of microbes to switch metabolic modes (e.g. respiration vs fermentation) that differ in yields, substrates, and excreted by-products as the environment changes.
**Stoichiometric matrix (S-matrix)**
A mathematical representation of the set of reactions that make up a metabolic network, with rows referring to metabolites and columns to reactions. Each entry specifies a stoichiometric coefficient. The $i,j$th entry of the stoichiometric matrix specifies the number of molecules of metabolite $i$ consumed per unit rate of reaction $j$.
**Community growth rate and its relation to natural selection for growth rate of single species**
We define the growth rate of the microbial community as $d \ln X_{T}/dt=\mu _{C}$ with $X_{T}$ as the sum of the biomasses of the individual species, i.e. $X_{T}=\sum _{i} X_{i}$. We show in the text that the growth rate of the community always equals the averaged growth rate of the species, regardless of whether it is steady state or not; hence, $\mu _{C}=\sum _{i} \phi _{i} d\ln X_{i}/dt$ with $\phi _{i}=X_{i}/X_{T}$ defining the biomass fraction of species $i$. We are not convinced that the community growth rate is under direct natural selection. We rather argue that single genotypes are selected for and that those with a higher growth rate than their genetic variants fix. Thus, each resident species is competing with its own genetic variants (most fiercely) and with other species. The genetic variant with the highest specific growth rate wins this competition and fixes. Because its physiology is slightly different than the ancestor it outcompeted, the community conditions change slightly. This influences the competition and natural selection of all species and their genetic variants too. Because each genetic variants evolves now towards a higher growth rate such that the community growth will do so too, as its value equals the mean value of all growth rate. In this concept, the abundance of species $i$, $X_{i}$, then equals the sum of the biomasses of all its genetic variants.

An example of a macrochemical reaction for the anaerobic growth of *S. cerevisiae* on glucose with ethanol as the byproduct [16] is given by,


\begin{eqnarray*} & 1.19 {\mathrm{C}_{6}\mathrm{H}_{12}\mathrm{O}_{6}+0.2 \, \mathrm{NH}_{4}^{+}+1.63 \, \mathrm{H}_{2}\mathrm{O}} \rightarrow \nonumber \\ &1 \, \mathrm{C-mol} \, {\mathrm{CH}_{1.8}\mathrm{O}_{0.5}\mathrm{N}_{0.2}+} 2.03 \, {\mathrm{C}_{2}\mathrm{H}_{6}\mathrm{O}+2.08 \, \mathrm{HCO}_{3}^{-}+2.28 \, \mathrm{H}^{+}} \nonumber\end{eqnarray*}

The coefficient in front of glucose (${\mathrm{C}_{6}\mathrm{H}_{12}\mathrm{O}_{6}}$) specifies that 1.19 moles of glucose (denoted by G) is needed to make 1 C-mole of biomass (${\mathrm{CH}_{1.8}\mathrm{O}_{0.5}\mathrm{N}_{0.2}}$; denoted by X) and 2.03 moles of ethanol (${\mathrm{C}_{2}\mathrm{H}_{6}O}$; E) is made per C-mole of biomass. Thus, the yield (denoted by Y) of biomass on glucose equals $Y_{X/G}=1.19^{-1}$ C-mol/mol and the yield of ethanol on biomass equals $Y_{E/X}=2.03$ mol/C-mol. The chemical element composition of biomass can differ between microbial species [17].

When the macrochemical reaction is written for 1 unit of biomass, the rate of the macrochemical reaction is equal to the specific growth rate (or *per capita*) ($\mu$ in C-mol/(C-mol hr)) of the associated species. (In some literature, the specific growth rate is denoted by $q_{X}$ or $\lambda$.) The amount of biomass can also be quantified in terms of gram dry weight instead of C-mole. The specific glucose uptake rate $q_{G}$ (in mol glucose/(C-mol hr)) and the specific ethanol production rate $q_{E}$ (in mol ethanol/(C-mol hr)) are proportional to the growth rate, i.e. $q_{G}=Y_{X/G}^{-1}\mu$ and $q_{E}=Y_{E/X}\mu$. The proportionality constants are so-called yields denoted by $Y_{P/S}$ for the yield of product $P$ on substrate $S$. These yields are the coefficients in the macrochemical equation (see Table 1). Similar proportionality relations hold between the other compounds’ specific rates and growth rate. The net flux of glucose uptake and ethanol production by yeast, when it occurs at a biomass abundance $X$ (in C-moles or grams), equals $J_{G}=q_{G}X$ and $J_{E}=q_{E}X$, respectively.

### Microbial communities at steady state

Steady-state microbial communities are characterized by the constancy of: (i) all concentrations of the extracellular metabolites, including those turned over by the consortium members; (ii) consequently, the growth and metabolic rates of the species, which depend on those concentrations; (iii) the death rates of all the species, and (iv) all intracellular concentrations (e.g. of proteins and metabolic intermediates; to achieve a state of ‘balanced growth’). Balanced growth and steady-state communities can be experimentally achieved in the lab (e.g. continuous cultures [23] and approximated during the exponential phase in a batch cultivation [24]) and are approximated in applications such as in wastewater treatment [25].

We define the relative abundance $\phi _{i}$ (an intensive variable) of a species $i$ as its absolute abundance $X_{i}$ (in C-mole or grams; an extensive variable) divided by the total abundance of the community (an extensive variable), $X_{T}=\sum _{j=1}^{N_{S}} X_{j}$, consisting of $N_{S}$ species, i.e. $\phi _{i}=X_{i}/X_{T}$.

When the community is in steady state, all the relative abundances of the species remain constant. This implies that their rate of change equal zero. In the appendix (section: derivation of equation 1), we derive that this rate of change equals,


(1) \begin{eqnarray*}& \frac{d \phi_{i}}{dt}= \left(\frac{d \ln X_{i}}{dt} - \Big \langle \frac{d \ln X_{i}}{dt} \Big \rangle \right)\phi_{i}=0.\end{eqnarray*}

In this equation, $\langle \frac{d \ln X_{i}}{dt} \rangle$ represents the average rate of change of the log abundance of all the species defined as $\sum _{j=1}^{N_{S}} \phi _{j} \frac{d \ln X_{j}}{dt}$ which equals $d \ln X_{T}/dt$, which we shown in the Appendix. Thus, at steady state, all species obey the following relations,


(2) \begin{eqnarray*}& \frac{d \ln X_{i}}{dt} = \frac{d \ln X_{T}}{dt} = \Big \langle \frac{d \ln X_{i}}{dt} \Big \rangle=\mu_{C},\end{eqnarray*}

i.e. they all have the same net *per-capita* growth rate, which we shall refer to as the community growth rate $\mu _{C}$, this growth rate therefore equals the mean growth rate and the growth rate of the entire community. Earlier it was proven that a steady-state microbial community requires the equality of all net growth rates [26, 27]. This equation always applies, at any steady state, regardless of the occurrence of any other processes than growth that can alter the abundance of the species, such as their inflow, outflow or death. In many cases in nature, the net growth rate of a community may turn out to be zero and the nutrient fluxes sustain both the maintenance requirements of the species and counter their death rates.

### Consideration of dead biomass

The consideration of the death rates of microorganisms forces the consideration of growing biomass (active) and dead biomass (metabolically inactive, e.g. loss of membrane integrity). Thus, the total biomass of a community or any of its species equals the sum of the associated living and dead biomass. We do not consider the recycling (growth on) dead biomass, because we currently lack any understanding of the stoichiometry of that process. This we see as an important open problem in the field [28].

We denote the abundance of the metabolically active living biomass by ‘L’ and the dead biomass by ‘D’. The total abundance of species $i$ therefore equals $X_{i}=L_{i}+D_{i}$ and the total community biomass equals $X_{T}=\sum _{i=1}^{N_{S}} X_{i}=\sum _{i=1}^{N_{S}} L_{i} + \sum _{i=1}^{N_{S}} D_{i}$.

We model the rates of change of the living, dead, and total biomass of a species as,


(3) \begin{eqnarray*} \frac{d L_{i}}{dt}&=(\mu_{i}-d_{i})L_{i} \nonumber \\ \frac{d D_{i}}{dt}&=d_{i} L_{i} \nonumber \\ \frac{d X_{i}}{dt}&=\frac{d L_{i}}{dt}+\frac{d D_{i}}{dt}=\mu_{i}L_{i}\end{eqnarray*}

with $\mu _{i}$ and $d_{i}$, respectively, as the *per-capita* growth and death rate. An extension of the theory, for instance, by incorporating other processes such as dispersion or predation would happen at this stage.

We introduce additional abundance fractions that can be distinguished: the relative abundance of the living and dead biomass of a species with respect to the total community biomass (i.e. $L_{i}/X_{T}$ and $D_{i}/X_{T}$) and with respect to the abundance of the species (i.e. $L_{i}/X_{i}$ and $D_{i}/X_{i}$). In the Appendix, we show that the latter species-normalized abundances relate to the growth rate and the death rates of the species in the following manner,


(4) \begin{eqnarray*}& \frac{L_{i}}{X_{i}}=1-\frac{d_{i}}{\mu_{i}}=\frac{\mu_{C}}{\mu_{i}}, \, \frac{D_{i}}{X_{i}}=\frac{d_{i}}{\mu_{i}}=1-\frac{\mu_{C}}{\mu_{i}}.\end{eqnarray*}

Thus, the death and living fraction of a species in a steady-state community can always be calculated from its total abundance using its growth rate and death rate. Additionally, $0<L_{i}/X_{i}<1$ and $0<D_{i}/X_{i}<1$ such that $0<d_{i}/\mu _{i}<1$.

From equations 2–4, we conclude that $d \ln X_{i} / dt = \mu _{i}-d_{i}=\mu _{C}$. This means that the death rate of a species can be determined from its (living and dead) fractions and the community growth rate.

### Characteristics of a steady-state microbial community

Summarizing the results from the previous section, a steady-state microbial community is characterized by the following properties:

1. The growth rate of the entire community equals $d \ln X_{T}/dt = \mu _{C}$.
2. The concentrations of the exchanged nutrients are constant. Under these conditions, the growth and death rates of all species are also constant, and they satisfy $\mu _{C} = \mu _{i} - d_{i} = \langle \mu - d \rangle$ (Equation (2)).
3. Each species has a living and a dead fraction, given by $L_{i}/X_{i} = 1 - \mu _{i}/d_{i} = \mu _{i} / \mu _{C}$ and $D_{i}/X_{i} = 1 - L_{i}/X_{i}$.
4. The ratio of the living to dead abundance of a species equals $L_{i} / D_{i} = \mu _{C} / d_{i}$.

These relations are useful for determining the relative abundances of living and dead biomass from experimental data, with, for instance, a comparison of a cell count from a flow cytometer (counting dead and live cells) and colony counting by plating (counting live cells).

Without considering the macrochemical equations of growth for all species, it is not possible to determine the relative abundances $X_{i} / X_{T}$ (i.e. $\phi _{i}$). In the next sections, we address this for different microbial communities. We start with a simple example. Additional examples dealing with communities having different topologies, including cycles and branches, and detailed derivations are provided in the Appendix.

### The relative abundances of ethanol-exchanging yeast and Acetobacter in a 2-species community

**Figure 1:**
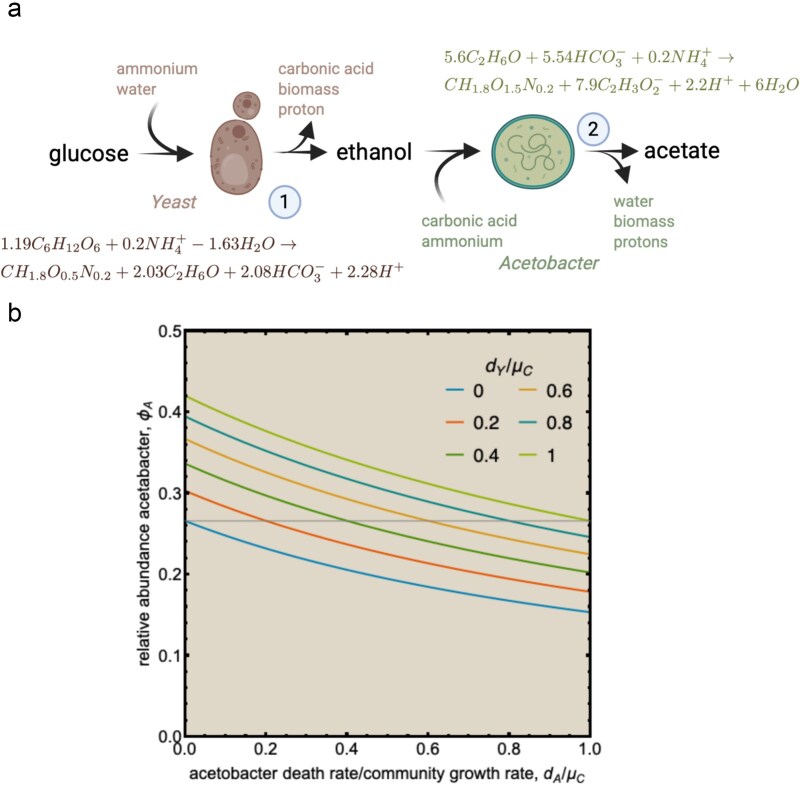
A 2-species community composed of a yeast and *Acetobacter* converting glucose into acetate, via ethanol. (A) The metabolic network formed by the cross-feeding of ethanol by a yeast and *Acetobacter* strain is shown together with macrochemical equations, which were obtained from Smeaton & Van Cappellen [22]. (B) The fraction of *Acetobacter* is shown as function of its normalized death rate.

To illustrate the procedure for expressing the relative abundances of species in terms of the yields of their metabolisms (i.e. their macrochemical equations), we consider a 2-species community responsible for wine turning sour [29]. It consists of a yeast producing ethanol from glucose and an *Acetobacter* converting the ethanol into acetic acid (Fig. 1A).

At steady state, the ethanol production rate by the yeast ($J_{Y,E}$) equals the ethanol consumption rate by the *Acetobacter* ($J_{A,E}$), i.e. $J_{Y,E} = J_{A,E}$. The net growth rates of both species equal the community growth rate:


\begin{align*} & \frac{d\ln X_{Y}}{dt} = \mu_{Y} - d_{Y} = \frac{d\ln X_{A}}{dt} = \mu_{A} - d_{A} = \mu_{C}. \end{align*}

In a batch-cultivation scenario, the biomasses $X_{Y}$, $X_{A}$, and $X_{T}$ would all increase exponentially with rate constant $\mu _{C}$. In a continuous culture (e.g. a chemostat), these rates would be set by the dilution rate, and biomass abundances would remain constant. The derivations that follow apply to all balanced-growth states of this community, regardless of the cultivation method.

The production rate $J_{Y,E}$ of ethanol ($E$) by the yeast ($Y$) is given by


(5) \begin{eqnarray*} J_{Y,E} &= q_{Y,E} X_{Y} = \frac{q_{Y,E}}{\mu_{Y}} \mu_{Y} X_{Y} \nonumber \\ &= Y_{E/X_{Y}} \, \mu_{Y} X_{Y} = Y_{E/X_{Y}} (\mu_{C} + d_{Y}) X_{Y},\end{eqnarray*}

where $Y_{E/X_{Y}}$ is the stoichiometric coefficient of ethanol in the yeast’s macrochemical growth equation (2.03 in Fig. 1A).

Similarly, the consumption rate $J_{A,E}$ of ethanol by the *Acetobacter* ($A$) is


(6) \begin{eqnarray*} J_{A,E} &= q_{A,E} X_{A} = \frac{q_{A,E}}{\mu_{A}} \mu_{A} X_{A} \nonumber \\ &= Y_{X_{A}/E}^{-1} \, \mu_{A} X_{A} = Y_{X_{A}/E}^{-1} (\mu_{C} + d_{A}) X_{A},\end{eqnarray*}

where $Y_{X_{A}/E}^{-1}$ is the stoichiometric coefficient of ethanol (5.6 in Fig. 1) in the macrochemical growth equation for *Acetobacter*.

We could alternatively define $J_{Y,E} = q_{Y,E} L_{Y}$ instead of $J_{Y,E} = q_{Y,E} X_{Y}$, and similarly for *Acetobacter*. This would require expressing the specific production and consumption rates in terms of living biomass only. However, because living biomass is often not directly measured in experimental settings, we use total biomass here. This choice does not qualitatively change the following derivations.

At steady state, the ethanol production rate by the yeast balances the consumption rate by the *Acetobacter*:


(7) \begin{eqnarray*}& Y_{E/X_{Y}} (\mu_{C} + d_{Y}) X_{Y} = Y^{-1}_{X_{A}/E} (\mu_{C} + d_{A}) X_{A}.\end{eqnarray*}

Dividing by the total abundance $X_{T} = X_{A} + X_{Y}$ gives an equation in terms of the relative abundances $\phi _{Y} = X_{Y} / X_{T}$ and $\phi _{A} = X_{A} / X_{T}$. Because these sum to one ($\phi _{Y} + \phi _{A} = 1$), the expression can be solved for the relative abundance of either species. For *Acetobacter*:


(8) \begin{eqnarray*} \phi_{A} = 1 - \phi_{Y} = \frac{Y_{E/X_{Y}} \left( 1 + \frac{d_{Y}}{\mu_{C}} \right)} {Y_{E/X_{Y}} \left( 1 + \frac{d_{Y}}{\mu_{C}} \right) + Y^{-1}_{X_{A}/E} \left( 1 + \frac{d_{A}}{\mu_{C}} \right)}.\end{eqnarray*}

We can parameterize equation (8) using $Y_{E/X_{Y}} = 2.03$ and $Y^{-1}_{X_{A}/E} = 5.6$, obtained from the macrochemical growth equations of the two microbes (Fig. 1A). This parameterization allows us to predict the relative abundance of *Acetobacter* as a function of its death rate, normalized by the community growth rate (Fig. 1B). The prediction aligns with intuition: the relative abundance of *Acetobacter* decreases as its own death rate increases, and increases when the death rate of the yeast rises.

### Insights into the role of the death rate

Equation (8), or equivalently


\begin{align*} & \frac{\phi_{A}}{\phi_{Y}} = \frac{Y_{E/X_{Y}} (\mu_{C} + d_{Y})} {Y^{-1}_{X_{A}/E} (\mu_{C} + d_{A})}, \end{align*}

shows that, for a given community growth rate $\mu _{C}$, an increase in the *Acetobacter* death rate $d_{A}$ would require a higher *Acetobacter* growth rate $\mu _{A} = \mu _{C} + d_{A}$, yet its relative abundance would still decrease. Conversely, a higher yield of ethanol per unit yeast biomass ($Y_{E/X_{Y}}$) or a higher yield of *Acetobacter* biomass per unit ethanol ($Y_{X_{A}/E}$) would increase the *Acetobacter* fraction.

So far, we have considered communities with $\mu _{C}> 0$, where $\mu _{i}> d_{i}$ for all species. In the special case $\mu _{C} = 0$, the growth and death rates are equal for each species ($\mu _{i} = d_{i}$), leading to a stationary biomass despite ongoing metabolic activity.

### Growth conditions and nutrient limitation

The growth conditions for glucose and acetate must also be considered.

First, consider a batch cultivation in which all nutrients (including glucose and ammonium) are initially in great excess. As the culture proceeds, nutrients are gradually depleted, and the two microbes can reach a quasi-steady state together, as described in the previous section.

Alternatively, in a continuous culture such as a chemostat, all nutrients flow in and out at a dilution rate $D$ [23]. In this case, the effective loss rates become $d_{Y} = D + d^{\prime}_{Y}$ and $d_{A} = D + d^{\prime}_{A}$, where $d^{\prime}_{Y}$ and $d^{\prime}_{A}$ are the intrinsic death rates of yeast and *Acetobacter*, respectively. The community growth rate is then $\mu _{C} = 0$, with $\mu _{Y} = D + d^{\prime}_{Y}$ and $\mu _{A} = D + d^{\prime}_{A}$. Both species must grow faster than the dilution rate to persist, and they will not grow at equal rates if their intrinsic death rates differ.

Both species consume ammonium (as evident from their macrochemical equations in Fig. 1), but this does not necessarily imply direct competition for ammonium. For example, if glucose is the limiting nutrient for the yeast and ethanol is limiting for the *Acetobacter*, then their growth rates are determined by the concentrations of these respective energy sources. This occurs when the concentrations of glucose and ethanol are close to their Monod constants [24], whereas the ammonium concentration is much higher than its Monod constant and thus in excess. In such cases, the community is limited by the energy sources rather than by nitrogen availability.

### Consideration of maintenance requirements

Pirt’s interpretation of maintenance energy requirements [18] can also be incorporated into the equations. It accounts for a growth-rate independent ATP requirement, a growth-independent, energy-source requirement. We assume that the growth-rate dependent requirement is already considered in the biomass yield on the energy source. For a more detailed discussion, we refer to Beeftink *et al*. [30].

We start from the steady-state requirement that the ethanol production flux equals the ethanol consumption flux:


(9) \begin{eqnarray*} q_{E,Y} X_{Y} = \underbrace{\left[ Y^{-1}_{X_{A}/E} (\mu_{C} + d_{A}) + m_{S,A} \right]}_{q_{E,A}} X_{A},\end{eqnarray*}

where $m_{S,A}$ is the maintenance requirement of *Acetobacter*, and $q_{E,A} = Y^{-1}_{X_{A}/E} \mu _{A} + m_{S,A}$ according to Pirt’s interpretation.

The maintenance requirement of yeast enters via the biomass yield of yeast on glucose, $Y_{X_{Y}/G}$:


(10) \begin{eqnarray*} q_{E,Y} X_{Y} &= \frac{q_{E,Y}}{q_{G,Y}} \, q_{G,Y} X_{Y} \nonumber \\ &= Y_{E/G} \left[ Y^{-1}_{X_{Y}/G} (\mu_{C} + d_{Y}) + m_{S,Y} \right] X_{Y},\end{eqnarray*}

where $m_{S,Y}$ is the maintenance requirement of yeast and $q_{G,Y} = Y^{-1}_{X_{Y}/G} \mu _{Y} + m_{S,Y}$ is Pirt’s equation for glucose uptake.

Combining these maintenance-extended expressions yields the following relation for the relative abundance of *Acetobacter*:


(11) \begin{eqnarray*} \phi_{A} = \frac{ Y_{E/G} \left[ Y^{-1}_{X_{Y}/G} \left( 1 + \frac{d_{Y}}{\mu_{C}} \right) + \frac{m_{S,Y}}{\mu_{C}} \right] } { Y^{-1}_{X_{A}/E} \left( 1 + \frac{d_{A}}{\mu_{C}} \right) + \frac{m_{S,A}}{\mu_{C}} + Y_{E/G} \left[ Y^{-1}_{X_{Y}/G} \left( 1 + \frac{d_{Y}}{\mu_{C}} \right) + \frac{m_{S,Y}}{\mu_{C}} \right] }.\end{eqnarray*}

This expression reduces to equation (8) when the maintenance requirements are zero. Maintenance requirements influence relative abundances in a manner similar to death rates: increasing the maintenance requirement of a species decreases its relative abundance.

### Net conversion of a yeast–*Acetobacter* community

At steady state, the net growth rates of yeast ($\mu _{Y} - d_{Y}$) and *Acetobacter* ($\mu _{A} - d_{A}$) both equal the community growth rate $\mu _{C}$. The rates of the macrochemical reactions for yeast and *Acetobacter* are therefore $\mu _{Y} X_{Y}$ and $\mu _{A} X_{A}$, respectively.

For each reactant in the two macrochemical equations, the net production or consumption rate by the community equals the reaction rate multiplied by the stoichiometric coefficient of that reactant. For ethanol, these net rates cancel (produced by yeast, consumed by *Acetobacter*). For other reactants, such as glucose, acetate, biomass, and ammonium, there is a net community-level production or consumption at steady state. The stoichiometry of the entire community at steady state is described by the macrochemical equation of the community, which we call the ‘community conversion’. This can be obtained directly from the macrochemical equations of the two species.

The derivation proceeds as follows. The two macrochemical equations occur at rates $\mu _{Y} X_{Y}$ and $\mu _{A} X_{A}$. For example, the net consumption rate of ammonium by the community is:


\begin{align*} & - Y_{N/Y} \, \mu_{Y} X_{Y} \;-\; Y_{N/A} \, \mu_{A} X_{A}. \end{align*}

This rate must equal the rate of the community conversion ($\mu _{C} X_{T}$) multiplied by the stoichiometric coefficient of ammonium in that equation ($Y_{N/C}$):


\begin{align*} & - Y_{N/Y} \, \mu_{Y} X_{Y} \;-\; Y_{N/A} \, \mu_{A} X_{A} \;=\; Y_{N/C} \, \mu_{C} X_{T}. \end{align*}

Thus, the ammonium coefficient in the community macrochemical equation is:


\begin{align*} & Y_{N/C} = - Y_{N/Y} \frac{\mu_{Y}}{\mu_{C}} \phi_{Y} - Y_{N/A} \frac{\mu_{A}}{\mu_{C}} \phi_{A}. \end{align*}

The same logic applies to all reactants in the net conversion equation. Cross-fed metabolites cancel out, and biomass terms require incorporating death rates.

In general, the macrochemical equation for the entire community (MEQ$_{C}$) can be expressed in terms of the species-level macrochemical equations (${\mathrm{MEQ}}_{Y}$ and ${\mathrm{MEQ}}_{A}$) as:


(12) \begin{eqnarray*} \frac{{\text MEQ}_{C}}{X_{T} {\mu}_{C}} &= {\text MEQ}_{Y} \frac{\mu_{Y}}{\mu_{C}} {\phi}_{Y} + {\text MEQ}_{A} \frac{\mu_{A}}{\mu_{C}} {\phi}_{A} \nonumber \\ &\quad - {\text CH_{1.8}O_{1.5}N_{0.2}} \frac{\text d_{Y}}{\mu_{\text C}} {\phi}_{Y} - {\text CH_{1.8}O_{1.5}N_{0.2}} \frac{\text d_{A}}{\mu_{\text C}} {\phi}_{\mathrm{A}}.\end{eqnarray*}

When the death rates are negligible, this reduces to the following macrochemical reaction for the community:


(13) \begin{eqnarray*} 0.9 \, {\mathrm{C}_{6}\mathrm{H}_{12}\mathrm{O}_{6} + 0.2 \, \mathrm{NH}_{4}^{+}} &\rightarrow{\mathrm{CH}_{1.8}O_{1.5}\mathrm{N}_{0.2} + 2.1 \, \mathrm{C}_{2}\mathrm{H}_{3}\mathrm{O}_{2}^{-}} \nonumber \\ &\quad + 2.2 \, {\mathrm{H}^{+} + 0.05 \, \mathrm{HCO}_{3}^{-} + 0.4 \, \mathrm{H}_{2}O}.\end{eqnarray*}

This equation constitutes the net conversion of the community—its ‘ecological service’.

A more general and compact derivation of the community net conversion, applicable to any community, is provided in the Appendix using a matrix formulation.

### Stoichiometric explanation of relative species abundances in terms of ATP metabolism

In the yeast–*Acetobacter* community considered above, the biomass ratio is proportional to the product of two yield coefficients:


\begin{align*} & \frac{\phi_{A}}{\phi_{Y}} \propto Y_{X_{A}/E} \, Y_{E/X_{Y}}. \end{align*}

An increase in either coefficient changes the abundance ratio. For example, the yeast could increase $Y_{E/X_{Y}}$ by producing more ethanol per unit biomass. This yield depends on the ATP requirement for biomass synthesis via fermentation: the ATP needed to assemble all macromolecules from central carbon metabolism precursors, minus the ATP produced during precursor biosynthesis. A more detailed treatment of this is given in the Appendix.

To generalize these ideas, consider a theoretical three-species community that sequentially converts a high-energy substrate $S_{1}$ into a low-energy product $S_{4}$:


\begin{align*} & S_{1} \xrightarrow[]{\mathrm{microbe}_{1}} S_{2} \xrightarrow[]{\mathrm{microbe}_{2}} S_{3} \xrightarrow[]{\mathrm{microbe}_{3}} S_{4}. \end{align*}

Here $S_{1}$, $S_{2}$, and $S_{3}$ serve as both carbon and energy sources for the three species. For example, $S_{1}$ could be glucose, $S_{2}$ ethanol, $S_{3}$ acetate, and $S_{4}$ methane or carbon dioxide. Each substrate is partly converted into biomass and partly into the next catabolic product in the chain. Let $X_{1}$, $X_{2}$, and $X_{3}$ denote the biomasses of the three species.

At steady state, the production and consumption rates of $S_{2}$ and $S_{3}$ must balance. This yields:


\begin{align*} & \frac{X_{1}}{X_{2}} = \frac{Y_{S_{2}/X_{2}} \, \mu_{2}}{Y_{S_{2}/X_{1}} \, \mu_{1}}, \quad \frac{X_{2}}{X_{3}} = \frac{Y_{S_{3}/X_{3}} \, \mu_{3}}{Y_{S_{3}/X_{2}} \, \mu_{2}}. \end{align*}

Here $Y_{S_{2}/X_{1}}$ and $Y_{S_{3}/X_{2}}$ denote the amount of catabolic product formed per unit biomass, whereas $Y_{S_{2}/X_{2}}$ and $Y_{S_{3}/X_{3}}$ denote the amount of catabolic substrate required to form one unit of biomass.

All four yield coefficients can be linked to a common metabolic principle. A carbon–energy source (e.g. glucose) is used for:

1. Energy generation via ATP synthesis, and
2. Biosynthesis of precursors (e.g. pyruvate and glucose-6-phosphate) that are assembled into monomers for macromolecules (nucleic acids, amino acids, and lipids).

Precursor biosynthesis can generate ATP (e.g. pyruvate from glycolysis yields 2 ATP/glucose) or consume ATP (e.g. nucleotide synthesis from glucose-6-phosphate requires 1 ATP/glucose). The total ATP demand for biomass formation equals the ATP needed to convert precursors into macromolecules minus the ATP generated during precursor biosynthesis. The residual ATP demand must be met by catabolism, producing the next metabolite in the chain ($S_{2}$ for microbe 1, $S_{3}$ for microbe 2). The yields $Y_{S_{2}/X_{1}}$ and $Y_{S_{3}/X_{2}}$ are therefore determined by this ATP balance, which in turn controls the steady-state abundances of $X_{2}$ and $X_{3}$.

In summary, the yield coefficients in macrochemical equations reflect the intracellular balance between catabolic ATP yields and anabolic ATP demands. Fermentation-product yields can be interpreted at coarse-grained level from this perspective—known as the ‘ATP method’ for deriving macrochemical equations [31]. This framework directly links intracellular energy metabolism to relative species abundances in steady-state communities.

### Stoichiometric imbalance and spontaneous niche creation in simple cross-feeding communities

So far, we have only considered unidirectional cross-feeding. We now turn to bidirectional cross-feeding, where two species coexist and each produces a metabolite consumed by the other. We will show that stoichiometric analysis predicts that, in general, the synthesis and degradation rate of one of these cross-fed metabolites will not balance such that a net production of it occurs. This ‘imbalanced metabolite’ then attains a relative high steady-state concentration, maintained by its net production by the community and its diffusion rate into the medium (assumed infinite). [This steady state will always arise because the diffusion rate is a linear function of the diffusion metabolite (it is not saturable).] This imbalanced metabolite effectively creates a niche for a third species that can consume it to reduces its concentration (and its diffusion-escape rate to a negligible value). We refer to this phenomenon as ‘stoichiometric imbalance’. Although it is easiest to illustrate with two species, the same reasoning applies to many-species communities. Next, we will illustrate how this works.

Consider two species, $A$ and $B$: species $A$ produces metabolite $a$ and consumes metabolite $b$; species $B$ produces $b$ and consumes $a$. In addition to cross-feeding, we allow for outflow (escape by diffusion) of both $a$ and $b$ from the system. At steady state, the metabolite balances are:


\begin{eqnarray*} \frac{da}{dt} &=& 0 = J_{a,A} - J_{a,B} - J_{a}, \nonumber \\ \frac{db}{dt} &=& 0 = J_{b,B} - J_{b,A} - J_{b}, \nonumber \end{eqnarray*}

where $J_{a,A}$ is the production rate of $a$ by species $A$, $J_{a,B}$ is the consumption rate of $a$ by species $B$, and $J_{a}$ is the outflow rate (diffusion) of $a$ (similarly for $b$). When $J_{a}$ and $J_{b}$ both equal zero then $a$ and $b$ their production and consumption rates are both balanced and their diffusion (loss) rates into the medium are negligible. When this is not the case then either $a$ or $b$ escapes into the medium instead of being consumed by the corresponding species, this occurs when the production rate of $a$ or $b$ by, respectively, $A$ or $B$, exceeds its consumption rate by the other species. We will now show that one of the two metabolite will tend to diffuse away and create a niche for a third species.

Using the approach from previous sections, these fluxes can be expressed in terms of yields, growth rates, and biomass:


\begin{eqnarray*} 0 &=& \underbrace{Y_{a/A}(\mu_{C} + d_{A}) X_{A}}_{J_{a,A}} - \underbrace{Y^{-1}_{B/a}(\mu_{C} + d_{B}) X_{B}}_{J_{a,B}} - J_{a}, \nonumber \\ 0 &=& \underbrace{Y_{b/B}(\mu_{C} + d_{B}) X_{B}}_{J_{b,B}} - \underbrace{Y^{-1}_{A/b}(\mu_{C} + d_{A}) X_{A}}_{J_{b,A}} - J_{b}. \nonumber \end{eqnarray*}

Defining $X_{T} = X_{A} + X_{B}$, $\phi _{A} = X_{A} / X_{T}$, and $\phi _{B} = X_{B} / X_{T}$, the $a$-balance gives:


\begin{eqnarray*}& \phi_{A} = \frac{J_{a}/X_{T} + Y^{-1}_{B/a}(\mu_{C} + d_{B})} {Y_{a/A}(\mu_{C} + d_{A}) + Y^{-1}_{B/a}(\mu_{C} + d_{B})}, \nonumber\end{eqnarray*}

while the $b$-balance gives:


\begin{eqnarray*}& \phi_{A} = \frac{-J_{b}/X_{T} + Y_{b/B}(\mu_{C} + d_{B})} {Y^{-1}_{A/b}(\mu_{C} + d_{A}) + Y_{b/B}(\mu_{C} + d_{B})}. \nonumber\end{eqnarray*}

In steady state, these two expressions for $\phi _{A}$ must be equal.

Two cases arise:

- **Stoichiometrically balanced:**  $J_{a} = J_{b}$, which can include $J_{a} = J_{b} = 0$. In this special case, the elemental composition of $a$ and $b$ and the metabolic demands of $A$ and $B$ align perfectly, so neither metabolite accumulates. This is expected to be rare.
- **Stoichiometrically imbalanced:**  $J_{a} \neq J_{b}$, which we expect to be the general case. Here, one metabolite accumulates relative to the other.

In the imbalanced case, natural selection is expected to drive the community toward maximal growth rate. Under this condition, one overflow rate ($J_{a}$ or $J_{b}$) will approach zero. If $J_{a} = 0$, the system is $A$-limited, $J_{b}> 0$, and a niche exists for a third microbe that can grow on $b$. Conversely, if $J_{b} = 0$, the system is $B$-limited and a niche exists for a microbe that can grow on $a$.

In summary, stoichiometric reasoning alone predicts that bidirectional cross-feeding typically leads to the imbalanced production and diffusional loss of one cross-fed metabolite, thereby creating a new ecological niche.

### The carrying capacity and the net metabolic conversion of a large community

**Figure 2:**
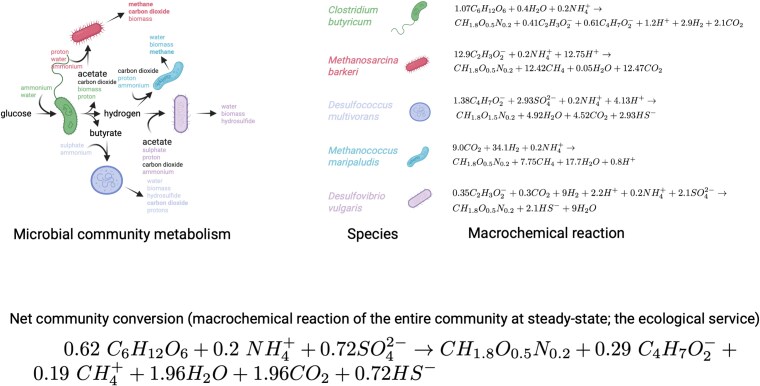
The metabolism and ecological service of a community of five microorganisms in terms of their macrochemical equations. The macrochemical equations of five microorganisms (from: Smeaton & Van Cappellen [22]) are shown that together convert glucose into methane, biomass, and carbon dioxide, via the metabolic intermediates: acetate, hydrogen, butyrate, and acetate. This net metabolic conversion of the microbial community, its ecological service, is shown as a macrochemical equation of the community. The ecological service can be computed from the relative abundances of the species and the macrochemical equations of the community using the method outlined in the main text. Chemical compounds: ${\mathrm{C}_{6}\mathrm{H}_{12}\mathrm{O}_{6}}$, (glucose), ${\mathrm{NH}_{4}^{+}}$ (ammonium), ${\mathrm{CH}_{1.8}\mathrm{O}_{0.5}\mathrm{N}_{0.2}}$ (biomass), ${\mathrm{CH}_{4}}$ (methane), ${\mathrm{H}_{2}\mathrm{O}}$ (water), ${\mathrm{CO}_{2}}$ (carbon dioxide), ${\mathrm{C}_{2}\mathrm{H}_{3}\mathrm{O}_{2}^{-}}$ (acetate), ${\mathrm{SO}_{4}^{3-}}$ (sulphate), and ${\mathrm{HS}^{-}}$ (hydrosulfide).

A community consisting of five microbial species converts glucose (e.g. deriving from plant litter) into carbon dioxide, methane, and biomass (Fig. 2). The species feed on each other’s waste products, via cross feeding of acetate, hydrogen, butyrate, and carbon dioxide. A representative macrochemical reaction of each of the microbial species is shown and were obtained from previously published data [22].

In the Appendix we provide a complete stoichiometric analysis of a microbial community performing anaerobic digestion (Fig. 2). We show how the species fractions can be expressed in terms of the stoichiometric coefficients of the macrochemical equations of the species, the net fluxes of nutrient in and out of the system, the community growth rate, and the death rates of all the species. This summarizes all the methods in a matrix formalism that is applicable to more complex cases then those considered so far.

This analysis also involves a numerical example, which leads to the following community conversion at steady state: glucose, ammonium, and sulphate are converted into biomass, butyrate, methane, carbon dioxide, and hydrosulfide,


(14) \begin{eqnarray*} && 0.72 \, {\mathrm{C}_{6}\mathrm{H}_{12}\mathrm{O}_{6}+0.2 \, \mathrm{NH}_{4}^{+}+0.72 \, \mathrm{SO}_{4}^{2-}}\nonumber \\ &&\rightarrow{\mathrm{CH}_{1.8}\mathrm{O}_{0.5}\mathrm{N}_{0.2} + 0.29 \, \mathrm{C}_{4}\mathrm{H}_{7}\mathrm{O}_{2}^{-}} \nonumber \\ &&\quad+0.19 \, {\mathrm{CH}_{4}^{+}+1.96 \, \mathrm{H}_{2}\mathrm{O}+1.96\mathrm{CO}_{2}+0.72 \, \mathrm{HS}^{-}}. \end{eqnarray*}

In this case, four of the five species *Clostridium butyricum, Desulfovibrio vulgaris, Desulfococcus multivorans*, and *Methanosarcina barkeri* are active at, respectively, the following biomass fractions:


\begin{align*} & \phi_{cb}=0.67, \, \phi_{dv}=0.22, \, \phi_{dm}=0.089, \, \phi_{mb}=0.015. \end{align*}

Glucose functions here as the energy source, and 0.72 mol glucose are required to form 1 C-mol of community biomass, as derived from the overall community stoichiometric equation. The carrying capacity of the environment equals therefore 1/0.72 mole biomass per mole glucose. This solution was obtained using community flux balance analysis [26], which calculates a stoichiometrically consistent flux distribution by maximizing the community growth rate under flux-balance constraints. This procedure involved a system of four flux-balance equations, which contains nine unknowns, i.e. five species fractions and four exchange fluxes (shown in the Appendix), and is therefore underdetermined. To obtain a solution, we applied linear programming and determined the values of the unknown variable that maximize the community growth rate. This simultaneously yields the optimal species fractions and the exchange fluxes. Because the community makes butyrate (and methane) a niche still exists for a butyrate (and methane) consumer to fill. For more information, consult the Appendix.

## Discussion

Microbial ecology is progressing towards a quantitative understanding of how microbial metabolism shapes community properties. Our current understanding is however less advanced than that of metabolism of single species, which involves enzyme biochemistry [32], the resulting physiology [8, 9], and outcomes of selective pressures [14]. A first step in that direction for microbial ecology is understanding the relationship between the metabolic stoichiometry of microbial growth (macrochemical equations), the relative abundance of species, and the net metabolic conversion of a steady-state community. Our framework links these quantities while accounting for maintenance, living and dead biomass, stoichiometric imbalance, and various community topologies (including those analysed in the Appendix).

Beyond providing a conceptual understanding, the balance equations derived here can be applied directly to experimental data. For example, relative abundances can be inferred from measured fluxes, especially when combined with biomass measurements and stable isotope probing (SIP) proteomics [33, 34]. SIP proteomics can help link specific metabolic activities to individual species, and the framework can be integrated with community flux balance analysis in cases where the system of equations is underdetermined due to limited data. Metabolite-flux and biomass measurements can also reveal the metabolic capacities of functional guilds. Their macrochemical equations may be selected from candidate equations and then the subset is chosen that fits the data best, using parameter estimation methods. Alternatively, all macrochemical equations are inferred from experimental data, provided it is sufficiently comprehensive. Finally, flux and biomass data are still rare as those measurement can only be done under controlled conditions and not directly for samples from nature. Then metagenomics is at the moment one of the most robust methods to determining the community composition and addressing its (metabolic) functionalities and estimating species fractions. In those circumstances, our method can be applied when the catabolic networks of the sequences species are inferred from gene analysis. This allows for the determination of the catabolic potential of the species, and its metabolic potential in terms of macrochemical equations can then be determined using previously described methods [17, 22].

Models such as generalized Lotka Volterra and resource–consumer models, do not necessarily consider stoichiometry. These phenomenological models are particularly useful for communities of poorly characterized species or when the focus is on abundance dynamics rather than precise metabolic activities—for instance, the dynamics of their abundances in terms of generalized species–species interactions [35]. Cases also exist where the interest is primarily in terms of the consequences of the metabolic capacities and interactions between the species. For example, how they concertedly maintain a particular metabolic ecological service such as, for instance, the anaerobic conversion of plant litter into greenhouse gases [6]. Then stoichiometric models are more relevant than more phenomenological models.

Stoichiometric models can describe certain phenomena better than non-stoichiometric models. A straightforward example occurs for the exchange of two nutrients $a$ and $b$ between two species $A$ and $B$. $A$ makes $a$ and feeds on $b$ made by $B$, which feeds on $a$. (For instance, $a$ is acetate and $b$ is an amino acid.) In such a cross-feeding exchange it will generally be so that the system settles to a steady state with equal specific growth rates of the two species and that one of the exchanged nutrients remains constant by a balanced production and consumption, whereas the other accumulates (stoichiometric imbalance; see Results section). This accumulation cannot be predicted by a non-stoichiometric model of the microbial community. Whether $a$ or $b$ accumulates depends on the precise stoichiometry of $A$’s and $B$’s macrochemical equation. If $a$ accumulates, then other species can invade the community then when $b$ accumulates. Stoichiometric models are needed to distinguish between these scenarios.

It is important to distinguish the framework presented in this work from community flux balance analysis (cFBA) (e.g. [26]). They are complementary. Whereas cFBA starts with genome-scale stoichiometric models of the metabolism of the species—comprising all metabolic capacities—and uses numerical optimization of an objective to find intracellular flux distributions that are consistent with balanced community growth, the present framework operates at the level of macrochemical equations per species and effectively assumes a metabolic behaviour of the species as a hypothesis or in agreement with experimental data. Our method then yields closed-form analytical expressions for species relative abundances, living/dead biomass fractions, community net conversion, and conditions for niche creation. These analytical results provide understanding, and they are not direct outputs of cFBA. The two approaches become particularly complementary when stoichiometric balance equations of the method explained in this paper are underdetermined, in which case cFBA can be used to resolve the remaining degrees of freedom, as in the five-species example of Fig. 2. Formally integrating these two levels of description by deriving MEQs directly from genome-scale networks and feeding community-level stoichiometric constraints back into cFBA is an interesting direction for future work.

Although we accounted for the presence of living and dead biomass, we did not model the recycling of dead biomass components. This extension was omitted because the stoichiometry of biomass recycling is currently too poorly understood, but it could be incorporated once better quantitative information becomes available [28]. Another important next development is the consideration of the adaptation dynamics of microbial communities, in terms of the adaptation strategies of individual microbial species, to the environment that they concertedly shape (ecological feedback) by exchanging chemical compounds and spatial organization.

## Supplementary Material

Appendix_wrag133

## Data Availability

This paper is theoretical and does not rely on any published data.

## References

[1] Falkowski PG, Fenchel T, Delong EF. The microbial engines that drive Earth’s biogeochemical cycles. *Science* 2008;320:1034–9. 18497287 10.1126/science.1153213

[2] Widder S, Allen RJ, Pfeiffer T, et al. Challenges in microbial ecology: building predictive understanding of community function and dynamics. *ISME J* 2016;10:2557–68. 10.1038/ismej.2016.45 27022995 PMC5113837

[3] Handelsman J . Metagenomics: application of genomics to uncultured microorganisms. *Microbiol Mol Biol Rev* 2004;68:669–85. 10.1128/MMBR.68.4.669-685.2004 15590779 PMC539003

[4] Tyson GW, Chapman J, Hugenholtz P, et al. Community structure and metabolism through reconstruction of microbial genomes from the environment. *Nature* 2004;428:37–43. 10.1038/nature02340 14961025

[5] Konopka A . What is microbial community ecology? *ISME J* 2009;3:1223–30. 10.1038/ismej.2009.88 19657372

[6] Goldford JE, Lu N, Bajić D, et al. Emergent simplicity in microbial community assembly. *Science* 2018;361:469–74. 10.1126/science.aat1168 30072533 PMC6405290

[7] Molenaar D, Berlo Rvan, Ridder Dde. Shifts in growth strategies reflect tradeoffs in cellular economics. *Mol Syst Biol* 2009;5:323. 10.1038/msb.2009.82 19888218 PMC2795476

[8] Scott M, Hwa T. Shaping bacterial gene expression by physiological and proteome allocation constraints. *Nat Rev Microbiol* 2023;21:327–42. 36376406 10.1038/s41579-022-00818-6 PMC10121745

[9] Bruggeman FJ, Planqué R, Molenaar D. Searching for principles of microbial physiology. *FEMS Microbiol Rev* 2020;44:821–44. 33099619 10.1093/femsre/fuaa034 PMC7685786

[10] O’Brien EJ, Monk JM, Palsson BO. Using genome-scale models to predict biological capabilities. *Cell* 2015;161:971–87. 26000478 10.1016/j.cell.2015.05.019 PMC4451052

[11] Mori M, Zhang Z, Banaei-Esfahani A, et al. From coarse to fine: the absolute *Escherichia coli* proteome under diverse growth conditions. *Mol Syst Biol* 2021;17:1–23. 10.15252/msb.20209536 PMC8144880 34032011

[12] Elsemman IE, Rodriguez Prado A, Grigaitis P, et al. Whole-cell modeling in yeast predicts compartment-specific proteome constraints that drive metabolic strategies. *Nat Commun* 2022;13:801. 35145105 10.1038/s41467-022-28467-6 PMC8831649

[13] Basan M, Hui S, Okano H, et al. Overflow metabolism in *Escherichia coli* results from efficient proteome allocation. *Nature* 2015;528:99–104. 10.1038/nature15765 26632588 PMC4843128

[14] De Groot DH, Van Boxtel C, Planqué R. The number of active metabolic pathways is bounded by the number of cellular constraints at maximal metabolic rates. *PLoS Comput Biol* 2019;15:e1006858. 30856167 10.1371/journal.pcbi.1006858 PMC6428345

[15] Pacheco AR, Moel M, Segrè D. Costless metabolic secretions as drivers of interspecies interactions in microbial ecosystems. *Nat Commun* 2019;10:103. 10.1038/s41467-018-07946-9 30626871 PMC6327061

[16] Heijnen JJ, Van Dijken JP. In search of a thermodynamic description of biomass yields for the chemotrophic growth of microorganisms. *Biotechnol Bioeng* 1992;39:833–58. 10.1002/bit.260390806 18601018

[17] Kleerebezem R, van LMCM. A generalized method for thermodynamic state analysis of environmental systems. *Crit Rev Environ Sci Technol* 2010;40:1–54. 10.1080/10643380802000974

[18] Pirt SJ . The maintenance energy of bacteria in growing cultures. *Proc R Soc Lond B Biol Sci* 1965;163:224–31. 10.1098/rspb.1965.0069 4378482

[19] Stein RR, Bucci V, Toussaint NC, et al. Ecological modeling from time-series inference: insight into dynamics and stability of intestinal microbiota. *PLoS Comput Biol* 2013;9:e1003388. 10.1371/journal.pcbi.1003388 24348232 PMC3861043

[20] Battley EHA . Theoretical study of the thermodynamics of microbial growth using *Saccharomyces cerevisiae* and a different free energy equation. *Q Rev Biol* 2013;88:69–96. 23909225 10.1086/670529

[21] Clement TJ, Baalhuis EB, Teusink B, et al. Unlocking elementary conversion modes: ecmtool unveils all capabilities of metabolic networks. Patterns, 2021;2:100177. 33511367 10.1016/j.patter.2020.100177 PMC7815953

[22] Smeaton CM, Van Cappellen P. Gibbs energy dynamic yield method (GEDYM): predicting microbial growth yields under energy-limiting conditions. *Geochim Cosmochim Acta* 2018;241:1–16.

[23] Novick A, Szilard L. Description of the chemostat. *Science* 1950;112:715–6. 10.1126/science.112.2920.715 14787503

[24] Monod J . The growth of bacterial cultures. *Ann Rev Microbiol* 1949;3:371–94. 10.1146/annurev.mi.03.100149.002103

[25] Henze M, Gujer W, Mino T, et al. Activated Sludge Models ASM1, ASM2, ASM2d and ASM3. London: IWA Scientific and Technical Report No. 9, IWA Publishing, 2000.

[26] Khandelwal RA, Olivier BG, Röling WFM. Community flux balance analysis for microbial consortia at balanced growth. *PLoS One* 2013;8:e64567. 10.1371/journal.pone.0064567 23741341 PMC3669319

[27] Powell GE . Equalisation of specific growth rates for syntrophic associations in batch culture. *J Chem Technol Biotechnol* 1984; 34:97–100.

[28] Simpson AJ, Simpson MJ, Smith E. Microbially derived inputs to soil organic matter: are current estimates too low? *Environ Sci Technol* 2007;41:8070–6. 10.1021/es071217x 18186339

[29] Bartowsky EJ, Henschke PA. Acetic acid bacteria spoilage of bottled red wine—a review. *Int J Food Microbiol* 2008;125:60–70. 10.1016/j.ijfoodmicro.2007.10.016 18237809

[30] Beeftink HH, Van der Heijden RTJM, Heijnen JJ. Maintenance requirements: energy supply from simultaneous endogenous respiration and substrate consumption. *FEMS Microbiol Ecol* 1990;6:203–9.

[31] Heijnen JJ, Roels JAA. Macroscopic model describing yield and maintenance relationships in microbial cultures. *Biotechnol Bioeng* 1981;23:739–63. 10.1002/bit.260230407

[32] Teusink B, Passarge J, Reijenga CA, et al. Can yeast glycolysis be understood in terms of in vitro kinetics of the constituent enzymes? *Testing biochemistry Eur J Biochem* 2000;267:5313–29. 10951190 10.1046/j.1432-1327.2000.01527.x

[33] Li Z, Yao Q, Guo X, et al. Genome-resolved proteomic stable isotope probing of soil microbial communities using CO2 and 13C-methanol. *Front Microbiol* 2019;10:2706. 10.3389/fmicb.2019.02706 31866955 PMC6908837

[34] Kleiner M, Dong X, Hinzke T, et al. Metaproteomics method to determine carbon sources and assimilation pathways of species in microbial communities. *Proc Natl Acad Sci USA* 2018;115:E5576–84. 10.1073/pnas.1722325115 29844191 PMC6004456

[35] Higgins FJ, Gore J. Community structure follows simple assembly rules in microbial microcosms. *Nat Ecol Evol* 2017;1:0109. 10.1038/s41559-017-0109 28812687

