# Supplementary material for: Stoichiometric analysis of microbial communities links function, structure, and biomass carrying capacity
Source: ISME J. 2026 Jul 3;20(1):wrag133. doi: 10.1093/ismejo/wrag133 (PMC13453314; doi:10.1093/ismejo/wrag133)
Supplement: Supplementary file 1 [file appendix_wrag133.pdf]

Appendix to: Stoichiometric analysis of microbial communities links function, structure, and biomass carrying capacity

Frank J Bruggeman[1,*], Timothy Paez-Watson[1,3], Bas Teusink[1], and Robbert Kleerebezem[2]

[1]Systems Biology, AIMMS, VU University, De Boelelaan 1108, 1081 HZ, Amsterdam, The Netherlands
[2]Environmental Biotechnology, TU Delft, Van der Maarsweg 9,2629 HZ, DelftThe Netherlands
[3]Laboratory of Systems and Synthetic Biology, Wageningen University and Research Centre, Stippeneng 4, 6708 WE, Wageningen, The Netherlands
[*]Corresponding author: f.j.bruggeman@vu.nl

1 Derivation of the macrochemical reaction of a steady-state metabolic network

Consider a metabolic network with stoichiometric matrix $\mathbf{N}$ at steady state such that its flux vector $\mathbf{J}$ obeys

$$\mathbf{NJ} = 0,$$

giving rise to as many steady-state flux balances as variable reactants in the networks (equal to the number of rows of $\mathbf{N}$). The rows of the stoichiometric matrix refers to all the reactant concentrations of this network that are considered variable. A steady state can be achieved by fixing the concentration of external metabolites which either flow in or out of the network. This effectively sets a free-energy potential that maintains the network in a non-equilibrium steady state involving constant mass and energy fluxes through the network.

The macrochemical equation (MEQ) that corresponds to this state of the metabolic network can be obtained from

$$\text{MEQ} = \mathbf{m}^T \mathbf{N}' \mathbf{J}$$

with: $\mathbf{m}$ is the vector with metabolite names (ordered according to the row ordering of the stoichiometric matrix) and $\mathbf{N}'$ is the matrix $\mathbf{N}$ augmented with stoichiometry of reactions involving the fixed external metabolites. The MEQ can be written in its macrochemical reaction form by adding all the negative terms left of a chemical reaction arrow and those that are positive to its right-hand side. If you would do this for glycolysis and the complete conversion of glucose into ethanol then one would consider the concentrations of glucose, ethanol, ATP, ADP and Pi fixed and the macrochemical equation would result in

$$-glucose - 2\ ADP - 2\ Pi + 2\ ATP + 2\ ethanol = 0.$$

The synthesis and degradation rates of all the other (variable) metabolites (including NADH and NAD) would all balance.

2 Derivation of equations appearing in the main text

2.1 Derivation of equation ??

Given $\phi_i(t) = X_i/X_T$, we obtain

$$\begin{aligned}
\frac{d\ln\phi_i}{dt} &= \frac{d\ln X_i}{dt} - \frac{d\ln X_T}{dt} \\
&= \frac{d\ln X_i}{dt} - \frac{1}{X_T}\frac{d}{dt}\sum_{i=1}^{N_S} X_i \\
&= \frac{d\ln X_i}{dt} - \sum_{i=1}^{N_S}\frac{1}{X_T}\frac{d}{dt}X_i \\
&= \frac{d\ln X_i}{dt} - \sum_{i=1}^{N_S}\phi_i\frac{d}{dt}\ln X_i \\
&= \frac{d\ln X_i}{dt} - \left\langle\frac{d}{dt}\ln X_i\right\rangle
\end{aligned}$$

with $\left\langle\frac{d}{dt}\ln X_i\right\rangle = \sum_{i=1}^{N_S}\phi_i\frac{d}{dt}\ln X_i$, i.e. the average $d\ln X_i/dt$. This derivation also shows that $\frac{d\ln X_T}{dt} = \left\langle\frac{d}{dt}\ln X_i\right\rangle$.

2.2 Derivation of equation 2

The relative abundances L_i/X_T, D_i/X_T and $X_i/X_T = L_i/X_T + D_i/X_T$ are all fixed at any steady state of the community. This implies the following relations:

$$\begin{aligned}
\frac{d}{dt}\ln\frac{L_i}{X_T} &= \frac{d}{dt}\ln L_i - \frac{d}{dt}\ln X_T = \mu_i - d_i - \frac{d}{dt}\ln X_T \\
\frac{d}{dt}\ln\frac{D_i}{X_T} &= \frac{d}{dt}\ln D_i - \frac{d}{dt}\ln X_T = d_i\frac{L_i}{D_i} - \frac{d}{dt}\ln X_T \\
\frac{d}{dt}\ln\frac{X_i}{X_T} &= \frac{d}{dt}\ln X_i - \frac{d}{dt}\ln X_T \\
&= \frac{1}{X_i}\frac{dL_i}{dt} + \frac{1}{X_i}\frac{dD_i}{dt} - \frac{d}{dt}\ln X_T \\
&= \mu_i\frac{L_i}{X_i} - \frac{d}{dt}\ln X_T.
\end{aligned}$$

Hence, the following relations hold for the community growth rate equals

$$\mu_C = \frac{d}{dt}\ln X_T = \mu_i - d_i = d_i\frac{L_i}{D_i} = \mu_i\frac{L_i}{X_i}. \quad (1)$$

From these relations, we can deduce that

$$\frac{L_i}{X_i} = \frac{\mu_C}{\mu_i} = 1 - \frac{d_i}{\mu_i},\ \frac{D_i}{X_i} = \frac{d_i}{\mu_i}. \tag{2}$$

These fractions sum to 1, because X_i is defined as the sum of $L_i + D_i$.

We already showed that the community growth rate μ_C equals $d \ln X_T/dt$ and the mean value of $d \ln X_i/dt$. An expression of this mean can be derived,

$$\begin{aligned}
\mu_C = \frac{d}{dt} \ln X_T &= \sum_i \frac{1}{X_T}\frac{dL_i}{dt} + \sum_i \frac{1}{X_T}\frac{dD_i}{dt} \\
&= \sum_i \frac{1}{X_T}(\mu_i - d_i)L_i + \sum_i \frac{1}{X_T} d_i L_i \\
&= \sum_i \frac{1}{X_T}\mu_i L_i \\
&= \sum_i \frac{X_i}{X_T}\mu_i \frac{L_i}{X_i} \quad \text{(using eq. 1)} \\
&= \sum_i \phi_i(\mu_i - d_i) \\
&= \langle \mu - d \rangle.
\end{aligned}$$

Thus, this last result and equation 1 indicate that the growth rate of each species equals, at any steady state of the community,

$$\mu_i = \mu_C + d_i,$$

which implies that the concentrations of the extracellular nutrients and products – of which some are toxins for other species – which impact the growth rate of species are eventually such that this relation holds. For example, in the simplest case the growth rate of a species depends only on the concentration of a single limiting nutrient concentration according to a Monod equation. Because the metabolic activity of a species is proportional to its growth rate and its energy-maintenance rate, the growth rate sets the net uptake and production rates of nutrients and products of one particular species. If a microbe cannot achieve the community growth rate it goes extinct.

Finally, using $\mu_C = \mu_i - d_i$ and equation 2, we conclude that

$$\frac{L_i}{X_i} = \frac{\mu_C}{\mu_i},\ \frac{D_i}{X_i} = 1 - \frac{\mu_C}{\mu_i}.$$

3 Derivation of the catabolic product and biomass yield expression in terms of ATP yields

We limit ourselves to a carbon source as energy source, from which a catabolic product is made as well as precursor molecules, consumed during the synthesis of amino acids, nucleic acids, and lipids.

Consider the following simplified representation of a 2-species community

$$CS \xrightarrow{\text{microbe 1}} CP \xrightarrow{\text{microbe 2}} .$$

CP is the catabolic product of microbe 1, made from its carbon source CS. CP is also the carbon source for microbe 2.

At steady state of the community, the net production and consumption rate of the catabolic product balances

$$q^1_{CP} X_1 = q^2_{CP} X_2 \quad \Rightarrow \quad \mu_1 Y^1_{CP/X_1} X_1 = \mu_2 Y^2_{CP/X_2} X_2$$

with $Y^1_{CP/X_1} = \frac{q^1_{CP}}{\mu_1}$ as the yield of catabolic product on biomass of microbe 1 (both made from CS) and $Y^2_{CP/X_2} = \frac{q^2_{CP}}{\mu_2}$, which is best represented as the inverse yield of biomass of microbe 2 on the catabolic product of microbe 1, i.e. $(Y^2_{X_2/CP})^{-1}$. These yields are net stoichiometric properties of the entire metabolic networks of these two microbes, are stoichiometric coefficients of the macrochemical reactions of the two species, and are each calculable with flux balance analysis from a genome-scale stoichiometric model of each of the species, from experiments, or from phenomenological models.

The last equation shows that

$$X_2 = Y^2_{X_2/CP} Y^1_{CP/X_1} \frac{\mu_1}{\mu_2} X_1 \tag{3}$$

and that therefore the biomass abundance of the second microbe depends on the yield of catabolic product on biomass of microbe 1 and the biomass yield of microbe 2 on this same compound. Next, it will be shown that both yields can be written in terms of the ATP metabolism of those two microbial species.

Next, we will express the yield of catabolic product on biomass Y^1_{CP/X_1} and the yield of biomass on a catabolic substrate $Y^2_{X_2/CP}$ in terms of the ratio of the amount of ATP produced during catabolic-product formation over the amount produced during precursor metabolism of both species.

The total specific carbon source flux uptake rate q_{CS}, in terms of CS for microbe 1 and in terms of CP for microbe 2, equals the sum of the carbon flux towards catabolic product (CP) and precursor (Pre) metabolism,

$$q_{CS} = q^{CS \to CP}_{CS} + q^{CS \to Pre}_{CS}.$$

Precursor metabolism involves the synthesis of metabolites from the carbon source that act as precursor for the biosynthesis of lipids, nucleic acids, and amino acids. For instance, alanine is made from the precursor metabolite pyruvate on glucose growth and yields 1 ATP per pyruvate, whereas the glucose-6-phosphate, a precursor for nucleic acid biosynthesis via the pentose phosphate pathway, requires 1 ATP per glucose-6-phosphate. When a precursor is made then no catabolic product is made (e.g. no ethanol, acetate, or formate, results from pyruvate when it is used as precursor). Catabolic-product formation is always accompanied by a net gain in ATP. The amount of catabolic product made depends on the ATP requirement that remains after subtraction of the ATP requirement per unit biomass by the amount made during precursor biosynthesis (this amount can be negative).

The ratio of carbon flow towards precursors versus catabolic product is defined by the following ratio of specific flux values,

$$\phi^{Pre/CP}_{CS} = \frac{q^{CS \to Pre}_{CS}}{q^{CS \to CP}_{CS}}.$$

Both microbes synthesize their ATP as part of the conversion of carbon source to catabolic product

$(CS \to CP)$ and during precursor formation from carbon source $(CS \to Pre)$,

$$\begin{aligned} q_{ATP} &= q_{ATP}^{CS\to CP} + q_{ATP}^{CS\to Pre} \\ &= n_{CS\to CP}^{ATP} q_{CS}^{CS\to CP} + n_{CS\to Pre}^{ATP} q_{CS}^{CS\to Pre} \end{aligned} \quad (4)$$

The specific production rate of catabolic product is defined by,

$$q_{CP} = n_{CS}^{CP} q_{CS}^{CS\to CP}.$$

The "n" stoichiometric coefficients are properties of the metabolic pathways that two microbes use to make catabolic products and precursor metabolites from catabolic substrates.

Next, we need to determine $Y_{X_2/CP}^2$, which is a yield of biomass of microbe 2 on its carbon source,

$$Y_{X/CS} = \frac{\mu}{q_{CS}} = \frac{\mu}{q_{ATP}} \frac{q_{ATP}}{q_{CS}} = Y_{X/ATP} \frac{q_{ATP}}{q_{CS}} \quad (5)$$

We assume that we know the yield of biomass on ATP (i.e. $Y_{X/ATP}$, which is also quite conserved across microbes), and quantifies how much ATP is needed to make 1 unit of biomass. Finally, we determine the yield of ATP per unit carbon source $\frac{q_{ATP}}{q_{CS}}$ in terms of the ratio of ATP made during precursor biosynthesis over that made during catabolic product formation,

$$\begin{aligned} \frac{q_{ATP}}{q_{CS}} &= \frac{n_{CS\to CP}^{ATP} q_{CS}^{CS\to CP} + n_{CS\to Pre}^{ATP} q_{CS}^{CS\to Pre}}{q_{CS}^{CS\to CP} + q_{CS}^{CS\to Pre}} \\ &= \frac{1 + \phi_{CS}^{Pre/CP} \frac{n_{CS\to Pre}^{ATP}}{n_{CS\to CP}^{ATP}}}{1 + \phi_{CS}^{Pre/CP}} n_{CS\to CP}^{ATP} \end{aligned} \quad (6)$$

We still need to determine the yield of catabolic product on biomass (i.e. for microbe 1: Y_{CP/X_1}^1.) The yield of a catabolic production of biomass can also be expressed in terms of whole-cell ATP stoichiometries,

$$Y_{CP/X} = \frac{q_{CP}}{\mu} = \frac{q_{CP}}{q_{ATP}} \frac{q_{ATP}}{\mu} = \frac{q_{CP}}{q_{ATP}} (Y_{X/ATP})^{-1}. \quad (7)$$

Next, we express $\frac{q_{CP}}{q_{ATP}}$ in terms of the stoichiometric coefficients of intracellular metabolism,

$$\begin{aligned} \frac{q_{CP}}{q_{ATP}} &= \frac{n_{CS}^{CP} q_{CS}^{CS\to CP}}{n_{CS\to CP}^{ATP} q_{CS}^{CS\to CP} + n_{CS\to Pre}^{ATP} q_{CS}^{CS\to Pre}} \\ &= \frac{1}{1 + \frac{n_{CS\to Pre}^{ATP}}{n_{CS\to CP}^{ATP}} \phi_{CS}^{Pre/CP}} \frac{n_{CS}^{CP}}{n_{CS\to CP}^{ATP}} \end{aligned} \quad (8)$$

With those four relations (eq. 5, 6, 7, and 8), we can express the ratio of biomass abundances in terms of the ATP-stoichiometries of the metabolism of the two species, by their substitution in equation 3,

$$\begin{aligned} X_2 &= Y_{X_2/CP}^2 Y_{CP/X_1}^1 \frac{\mu_1}{\mu_2} X_1 \\ &= \underbrace{Y_{X_2/ATP}^2 Y_{ATP/CP}^2}_{Y_{X_2/CP}^2, \text{eq.5}} \underbrace{Y_{CP/ATP}^1 (Y_{X_1/ATP}^1)^{-1}}_{Y_{CP/X_1}^1, \text{eq. 7}} \frac{\mu_1}{\mu_2} X_1 \\ &\propto \frac{1}{1 + \frac{n_{CS\to Pre}^{1,ATP}}{n_{CS\to CP}^{1,ATP}} \phi_{CS}^{1,Pre/CP}} \end{aligned} \quad (9)$$

$$\begin{pmatrix} Y_{cb}^{ac}\,\mu_{cb} & 0 & -\left(Y_{ac}^{dv}\right)^{-1}\mu_{dv} & 0 & -\left(Y_{ac}^{mb}\right)^{-1}\mu_{mb} \\ Y_{cb}^{h2}\,\mu_{cb} & -(Y_{h2}^{mm})^{-1}\,\mu_{mm} & -\left(Y_{h2}^{dv}\right)^{-1}\mu_{dv} & 0 & 0 \\ Y_{cb}^{bu}\,\mu_{cb} & 0 & 0 & -\left(Y_{bu}^{dm}\right)^{-1}\mu_{dm} & 0 \\ Y_{cb}^{co}\,\mu_{cb} & -(Y_{co}^{mm})^{-1}\,\mu_{mm} & -\left(Y_{co}^{dv}\right)^{-1}\mu_{dv} & Y_{dm}^{co}\,\mu_{dm} & Y_{mb}^{co}\,\mu_{mb} \\ 1 & 1 & 1 & 1 & 1 \end{pmatrix} \begin{pmatrix} \phi_{cb} \\ \phi_{mm} \\ \phi_{dv} \\ \phi_{dm} \\ \phi_{mb} \end{pmatrix} = \begin{pmatrix} J_{ac}/X_T \\ J_{h2}/X_T \\ J_{bu}/X_T \\ J_{co}/X_T \\ 1 \end{pmatrix}$$

Figure 1: Community balance relations in terms of species fractions $\boldsymbol{\phi}$ and biomass-normalised exchange fluxes J.

$$\mathbf{S} = \begin{bmatrix} -\left(Y_{glc}^{cb}\right)^{-1} & 0 & 0 & 0 & 0 & 0 & 0 & 0 & 0 & 0 & 0 & 0 & 0 & 0 \\ -\left(Y_{nh4}^{cb}\right)^{-1} & -(Y_{nh4}^{mm})^{-1} & -\left(Y_{nh4}^{dv}\right)^{-1} & -\left(Y_{nh4}^{dm}\right)^{-1} & -\left(Y_{nh4}^{mb}\right)^{-1} & 0 & 0 & 0 & 0 & 0 & 0 & 0 & 0 & 0 \\ 0 & Y_{mm}^{ch4} & 0 & 0 & Y_{mb}^{ch4} & 0 & 0 & 0 & 0 & 0 & 0 & 0 & 0 & 0 \\ 0 & 0 & Y_{dv}^{hs} & Y_{dm}^{hs} & 0 & 0 & 0 & 0 & 0 & 0 & 0 & 0 & 0 & 0 \\ 0 & 0 & -\left(Y_{so4}^{dv}\right)^{-1} & -\left(Y_{so4}^{dm}\right)^{-1} & 0 & 0 & 0 & 0 & 0 & 0 & 0 & 0 & 0 & 0 \\ Y_{cb}^{ac} & 0 & -\left(Y_{ac}^{dv}\right)^{-1} & 0 & -\left(Y_{ac}^{mb}\right)^{-1} & -1 & 0 & 0 & 0 & 0 & 0 & 0 & 0 & 0 \\ Y_{cb}^{h2} & -(Y_{h2}^{mm})^{-1} & -\left(Y_{h2}^{dv}\right)^{-1} & 0 & 0 & 0 & -1 & 0 & 0 & 0 & 0 & 0 & 0 & 0 \\ Y_{cb}^{bu} & 0 & 0 & -\left(Y_{bu}^{dm}\right)^{-1} & 0 & 0 & 0 & -1 & 0 & 0 & 0 & 0 & 0 & 0 \\ Y_{cb}^{co} & -(Y_{co}^{mm})^{-1} & -\left(Y_{co}^{dv}\right)^{-1} & Y_{dm}^{co} & Y_{mb}^{co} & 0 & 0 & 0 & -1 & 0 & 0 & 0 & 0 & 0 \\ 1 & 0 & 0 & 0 & 0 & 0 & 0 & 0 & 0 & -1 & 0 & 0 & 0 & 0 \\ 0 & 1 & 0 & 0 & 0 & 0 & 0 & 0 & 0 & 0 & -1 & 0 & 0 & 0 \\ 0 & 0 & 1 & 0 & 0 & 0 & 0 & 0 & 0 & 0 & 0 & -1 & 0 & 0 \\ 0 & 0 & 0 & 1 & 0 & 0 & 0 & 0 & 0 & 0 & 0 & 0 & -1 & 0 \\ 0 & 0 & 0 & 0 & 1 & 0 & 0 & 0 & 0 & 0 & 0 & 0 & 0 & -1 \end{bmatrix}$$

Figure 2: Stoichiometric matrix $\mathbf{S}$ for the community processes.

What becomes clear is that when microbe 1 meets its ATP demand for macromolecule biosynthesis from precursor molecules predominantly via precursor biosynthesis, hence $\phi_{CS}^{1,Pre/CP}$ is high, then it does not make much CP per unit biomass and, hence, the relative biomass abundance of species 2 is low. Thus, biomass abundance of the second species depends on the ATP stoichiometry of microbe 1 that makes the catabolic substrate of the microbe 2.

4 Illustration of the calculation of relative species abundances and community conversion

4.1 Calculation of the relative species abundances of a steady state community

The relative species abundances are determined from the steady-state condition of the metabolites that are exchanged between the species, the biomass and product yields of the species, and the in- and outflow of nutrients and products of the community. We will take anaerobic digestion of glucose by a microbial community (shown in figure 1 of the main text) as an example.

This community has four exchanged (variable) metabolites (i.e. acetate (ac), carbon dioxide (co), butyrate (bu) and hydrogen (h2); we consider glucose fixed) and five species (*Clostridium butyricum* (cb), *Methanosarcina barkeri* (mb), *Desulfococcus multivorans* (dm), *Methanococcus maripaludis* (mm), and *Desulfovibrio vulgaris* (dv)).

Because we have four variable metabolites, we have four flux balance equations that hold at steady state,

$$\begin{aligned}
q_{ac,cb}X_{cb} - q_{ac,dv}X_{dv} - q_{ac,mb}X_{mb} &= J_{ac} \\
q_{h2,cb}X_{cb} - q_{h2,mm}X_{mm} - q_{h2,dv}X_{dv} &= J_{h2} \\
q_{bu,cb}X_{cb} - q_{bu,dm}X_{dm} &= J_{bu} \\
q_{co,cb}X_{cb} - q_{co,mm}X_{mm} - q_{co,dv}X_{dv} + q_{co,dm}X_{dm} & \\
+q_{co,mb}X_{mb} &= J_{co}
\end{aligned}$$

The term on the left are the production and consumption rates of the species whereas the fluxes on the left (i.e. the J's) are the exchange fluxes with the environment. If glucose would be considered flowing into the environment, instead of fixed as it is now, then one more balance for glucose would have to be added.

Next, we introduce yields and relative species abundances. The yields are introduced by multiplying and dividing each term in the previous equations by the associated growth rate of the species, for example $q_{ac,cb}X_{cb} \to \frac{q_{ac,cb}}{\mu_{cb}}\mu_{cb}X_{cb} = Y^{ac}_{cb}\mu_{cb}X_{cb}$. The relative species abundances are introduced by division of all terms by the total biomass abundance X_T, For instance, $Y^{ac}_{cb}\mu_{cb}X_{cb} \to Y^{ac}_{cb}\mu_{cb}\frac{X_{cb}}{X_T} = Y^{ac}_{cb}\mu_{cb}\phi_{cb}$. The resulting set of equation can be couched in a matrix equation to which we also add the conservation relation for the species fractions (Figure 1).

This equation can be simplified by including the condition that the growth rate of each species equals the community growth rate (μ_C) plus the death rate of the species (e.g. $\mu_{cb} = \mu_C + d_{cb}$). We assume that we either know the growth rate of each of species or the community growth rate and the death rate of each species. From the macrochemical equations we obtain the numerical values of the yield coefficients. This allows for the determination of the species fractions by solving the previous set of linear equations.

4.2 Calculation of the community conversion (the ecological service) of a steady state community

To determine the community conversion – the macrochemical equation – of the steady-state community, we start from the steady-state flux vector $\mathbf{f}$ of the community,

$$\mathbf{f} = (\mu_{cb}\phi_{cb} \quad \mu_{mm}\phi_{mm} \quad \mu_{dv}\phi_{dv} \quad \mu_{dm}\phi_{dm} \quad \mu_{mb}\phi_{mb} \quad J_{ac} \quad J_{h2} \quad \dots \tag{10}$$

$$J_{bu} \quad J_{co} \quad d_{cb}\phi_{cb} \quad d_{mm}\phi_{mm} \quad d_{dv}\phi_{dv} \quad d_{dm}\phi_{dm} \quad d_{mb}\phi_{mb}) \tag{11}$$

We also require the names of all the nutrient concentrations and species abundances occuring in the system. These we collect in a vector ordered in the same order as the rows of the stoichiometric matrix. This vector we refer to as $\mathbf{n}$,

$$\mathbf{n} = (glc \quad nh4 \quad ch4 \quad h2 \quad so4 \quad ac \quad h2 \quad \dots \tag{12}$$

$$bu \quad co \quad X_{cb} \quad X_{mm} \quad X_{dv} \quad X_{dm} \quad X_{mb}) \tag{13}$$

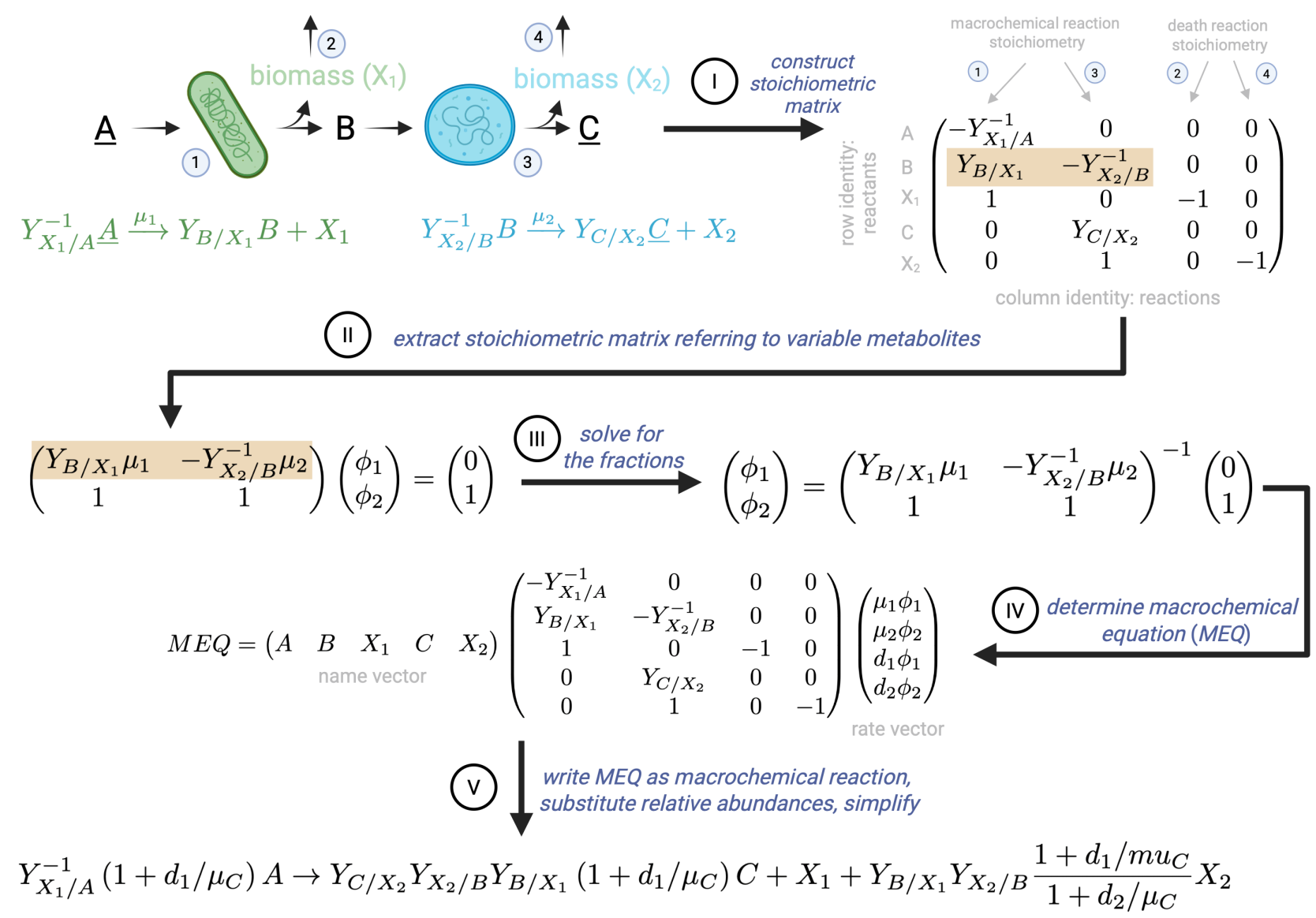

Figure 3: **Illustration of the derivation of the macrochemical reaction and carrying capacity of a 2-species community.** The underline indicates that we consider the concentration of the nutrients A and C to be fixed, to allow for the occurrence of a steady state. We assume that B does not overflow into the environment.

We also require the matrix $\mathbf{S}$ (Figure 2), containing the stoichiometric coefficients of all the processes that occur in the community.

The following multiplication then gives the macrochemical equation MEQ of the microbial community or the net conversion of the community.

$$MEQ = \mathbf{n}^T \mathbf{S} \mathbf{f}.$$

4.3 Numerical example of anaerobic digestion

The numerical example of the anaerobic digestion community and leading to the macrochemical equation (Figure 1 main text), corresponds to the following case. The relative abundances are:

$$\phi_{cb} = 0.67,\ \phi_{mm} = 0,\ \phi_{dv} = 0.22,\ \phi_{dm} = 0.089,\ \phi_{mb} = 0.015.$$

The fluxes are: $J_{glc,i} = 1$, $J_{nh4,i} = 0.28$, $J_{bu,o} = 0.40$, $J_{ch4,o} = 0.26$, and $J_{CO2,o} = 2.73$, $J_{H_2O,o} = 2.7$, $J_{HS,o} = 1$ and $J_{SO4,i} = 1$ (with "i" denoting inflow and "o" outtflow out of the community). This solution was obtained with linear programming (maximisation of the growth rate of the community).

5 Additional example analyses of small microbial communities

5.1 The carrying capacity and the net metabolic conversion for a 2-species community

The description of a two species community with the exchange of a single metabolite, similar to the yeast and *Acetobacter* community, can be done in general terms using two macrochemical reactions and two death processes (Figure 3). For simplicity, we only focus on the reactants that matter for the example (the nutrients A, B and C (e.g. glucose, ethanol and acetate) and the biomasses X_1 and X_2). Those could for instance be referring to the carbon (and energy) sources and products and do not consider a maintenance requirement.

The stepwise procedure for the determination of the community conversion of a community is shown starting from the macrochemical reactions of its species (Figure 3).

The first step is to construct the stoichiometric matrix of the metabolism of the community. This matrix has four columns corresponding to the two growth and two death reactions and five rows (i.e. the three nutrients and the two biomasses). The derivation of the community conversion requires first the derivation of the species fraction (step 2). This is done from the steady-state balances for the concentration of the variable nutrients in the network and the conservation relationship for the fraction. In this case we have only one balance equation for B. After the determination of the fractions, the community conversion is obtained by multiplying the stoichiometric matrix determined in step 1 from the left by the name vector of all the species and from the right by the steady-state flux vector. This equation can then be written as a reaction by gathering all the terms with a negative sign left of the reaction arrow and those with a positive sign to its right. In this way we obtain the stoichiometry of the net conversion of the community per unit X_1,

$$\begin{aligned} MEQ = & - Y_{X_1/A}^{-1} \left(1 + d_1/\mu_C\right) A + \\ & Y_{C/X_2} Y_{X_2/B} Y_{B/X_1} \left(1 + d_1/\mu_C\right) C + X_1 + \\ & Y_{B/X_1} Y_{X_2/B} \frac{1 + d_1/\mu_C}{1 + d_2/\mu_C} X_2 \end{aligned} \tag{14}$$

We consider this macrochemical reaction as the ecological service of the microbial community. This can, for instance, be the recycling of a chemical element or the conversion of plant litter into methane and carbon dioxide, which we work out in the Appendix. The community conversion (eq. 14) indicates that A is consumed to make C, X_1 and X_2. When the death rate of the first microbe would increase more A is consumed to produce more C and X_2 per unit X_1; so the ratio of X_2/X_1 increases. The carrying capacity of the community equals the yield coefficients in front of the biomasses (divided by the yield coefficient of A in case one would like to express the capacity per unit of the limiting nutrient).

When the community as a whole is not growing in size, i.e. $\mu_C = 0$, all microbes have a growth rate that equals their death rate. The macrochemical equation of the community then simplifies to

$$\begin{aligned} MEQ = & - Y_{X_1/A}^{-1} A + Y_{C/X_2} Y_{X_2/B} Y_{B/X_1} C + \frac{1}{2} X_1 + \\ & \frac{1}{2} Y_{B/X_1} Y_{X_2/B} X_2 \end{aligned} \tag{15}$$

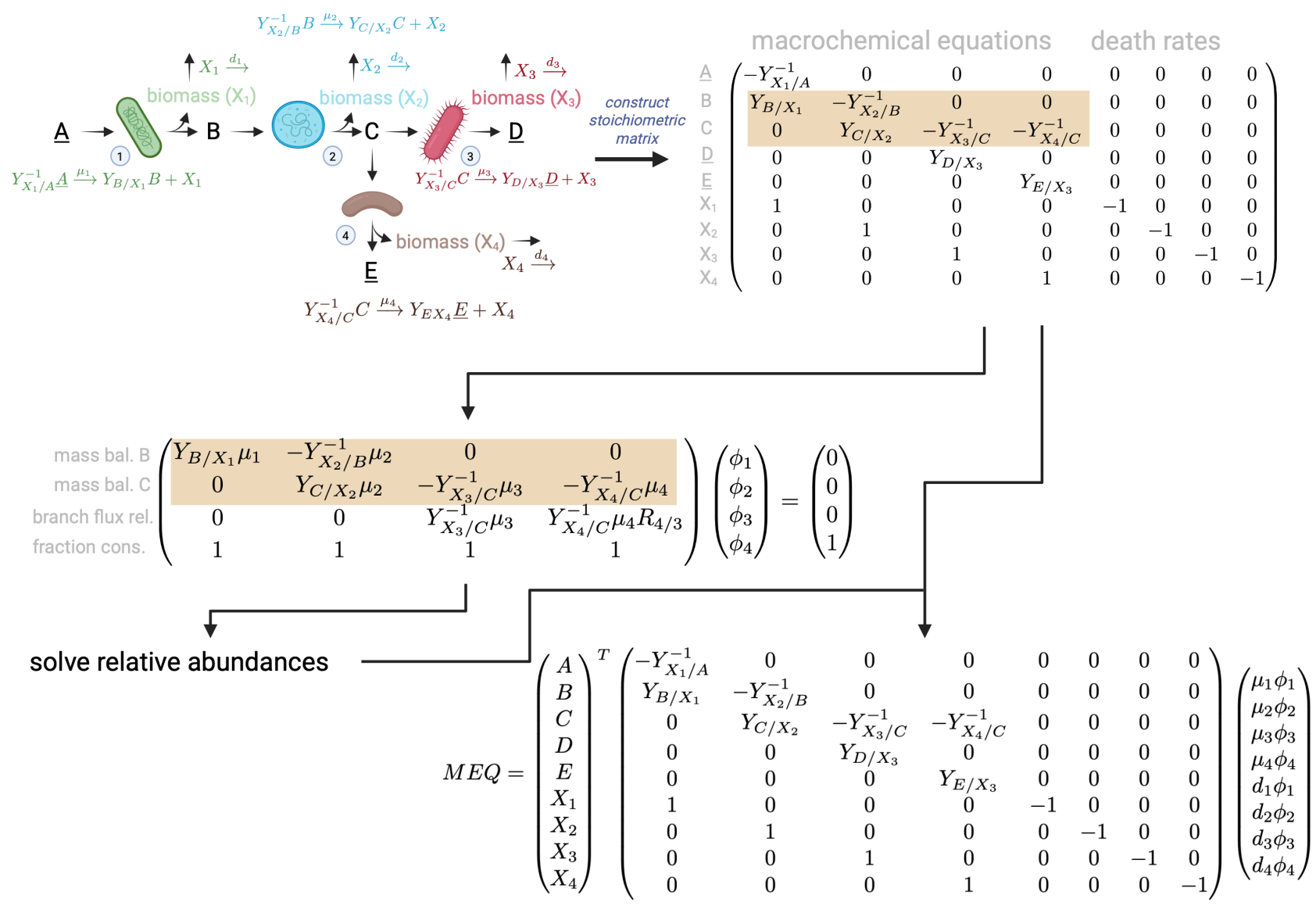

Figure 4: **Illustration of the derivation of the macrochemical reaction and carrying capacity of a 4-species and 1 branch community.** The underline indicates that we consider the concentration of the nutrients A, D and E to be fixed, to allow for the occurrence of a steady state. We assume that B and C do not overflow into the environment.

The community conversion will remain constant as long as none of the two species changes its metabolism in such a way that its macrochemical equation and net growth rate changes.

5.2 Branched microbial communities and branch flux ratios

Many microbial communities are branched and two or more species consume the same nutrient, which is the byproduct of the growth process of (an)other species (Figure 4). A branched network can be treated in a similar way as above, but because we end up with fewer exchanged metabolites between species than the number of species, we need to add more information. We will explicitly consider a small example to make this more clear. (The general case is treated in the Appendix in terms of some linear algebra properties of the stoichiometric matrix.)

We consider the following branched network composed out of 4 species and 2 exchanged nutrients,

$$\underline{A} \xrightarrow{\mu_1} B + X_1, \;\; X_1 \xrightarrow{d_1} \tag{16}$$

$$B \xrightarrow{\mu_2} C + X_2, \;\; X_2 \xrightarrow{d_2} \tag{17}$$

$$C \xrightarrow{\mu_3} \underline{D} + X_3, \;\; X_3 \xrightarrow{d_3} \tag{18}$$

$$C \xrightarrow{\mu_4} \underline{E} + X_4, \;\; X_4 \xrightarrow{d_4} \tag{19}$$

The following concentration balances exist for the two metabolic intermediates

$$\frac{dB}{dt} = J_{1,B} - J_{2,B}, \ \frac{dC}{dt} = J_{2,C} - J_{3,C} - J_{4,C} \tag{20}$$

Leading to the the following equations at steady state (using the same method outlined above),

$$0 = Y_{B/X_1}\mu_1\phi_1 - Y^{-1}_{X_2/B}\mu_2\phi_2 \tag{21}$$

$$0 = Y_{C/X_2}\mu_2\phi_2 - Y^{-1}_{X_3/C}\mu_3\phi_3 - Y^{-1}_{X_4/C}\mu_4\phi_4 \tag{22}$$

$$\phi_1 + \phi_2 + \phi_3 + \phi_4 = 1. \tag{23}$$

We aim to determine the four relative abundances. We are however one equation short (we have 4 ϕ's but only 3 equations that relate them) and require an additional relation between relative abundances to determine the relative abundances of the species. If we know (by measurement) the community's synthesis rate of D and E, then we know their ratio and obtain a new relation between relative abundances, i.e.

$$\frac{J_{4,C}}{J_{3,C}} = R_{4/3} = \frac{Y^{-1}_{X_3/C}\mu_3\phi_3}{Y^{-1}_{X_4/C}\mu_4\phi_4} \ \Rightarrow \phi_4 = \frac{Y^{-1}_{X_3/C}\mu_3}{Y^{-1}_{X_4/C}\mu_4 R_{4/3}}\phi_3 \tag{24}$$

This extra relation for the determination of all ϕ's form a set of linear equations. The associated equations are shown in the Appendix.

The net conversion of the community can again be calculated using the method explained in the previous section. It depends on the measured flux ratio and the ratio's of death rates and growth rates.

5.3 Cyclic microbial communities, chemical element conservation and a cycle law

Because many microbial communities conversions are associated with the recycling of chemical elements, we also consider a community that has a cyclic exchange of nutrients between species, driven by an external thermodynamic driving force. We consider a 4-species cycle where the first organism feeds on A, which is recycled by the fourth microbe,

$$\begin{aligned}
&A + \underline{E} \xrightarrow{\mu_1} X_1 + B, \ X_1 \xrightarrow{d_1} \\
&B \xrightarrow{\mu_2} X_2 + C, \ X_2 \xrightarrow{d_2} \\
&C \xrightarrow{\mu_3} X_3 + D, \ X_3 \xrightarrow{d_3} \\
&D \xrightarrow{\mu_4} X_4 + A, \ X_4 \xrightarrow{d_4}
\end{aligned} \tag{25}$$

As before, we do not show the other nutrients occurring in the macrochemical reaction which we assume fixed. In this case, we have four exchanged nutrients and species, corresponding to the following concentration balances,

$$\begin{aligned}
&\frac{dA}{dt} = J_4 - J_1, \ \frac{dB}{dt} = J_1 - J_2, \\
&\frac{dC}{dt} = J_2 - J_3, \ \frac{dD}{dt} = J_3 - J_4
\end{aligned} \tag{26}$$

Because the sum of these four equations equals 0, we have to conclude that the following sum of concentrations is conserved such that at all times the following relation holds: $T = A + B + C + D$.

Due to this linear dependency between the concentration balances, we only have three independent steady-state linear relations for the fractional abundances and their summation to 1,

$$0 = Y_{A/X_4}\mu_4\phi_4 - Y^{-1}_{X_1/A}\mu_1\phi_1 \tag{27}$$

$$0 = Y_{B/X_1}\mu_1\phi_1 - Y^{-1}_{X_2/B}\mu_2\phi_2 \tag{28}$$

$$0 = Y_{C/X_2}\mu_2\phi_2 - Y^{-1}_{X_3/C}\mu_3\phi_3 \tag{29}$$

$$1 = \phi_1 + \phi_2 + \phi_3 + \phi_4. \tag{30}$$

This set of equations is solvable and gives rise to the following abundance ratios

$$\frac{\phi_2}{\phi_1} = Y_{B/X_1}Y_{X_2/B}\frac{\mu_1}{\mu_2} \tag{31}$$

$$\frac{\phi_3}{\phi_2} = Y_{C/X_2}Y_{X_3/C}\frac{\mu_2}{\mu_3} \tag{32}$$

$$\frac{\phi_4}{\phi_3} = Y_{D/X_3}Y_{X_4/D}\frac{\mu_3}{\mu_4} \tag{33}$$

We can also deduce that because $0 = Y_{A/X_4}\mu_4\phi_4 - Y^{-1}_{X_1/A}\mu_1\phi_1$, the following relation holds as well

$$\frac{\phi_1}{\phi_4} = Y_{A/X_4}Y_{X_1/A}\frac{\mu_4}{\mu_1} \tag{34}$$

Because this equation needs to be in agreement with the previous abundance ratios, we deduce from the previous derived ratios that

$$\frac{\phi_4}{\phi_1} = \frac{\phi_4}{\phi_3}\frac{\phi_3}{\phi_2}\frac{\phi_2}{\phi_1} = Y_{B/X_1}Y_{X_2/B}Y_{C/X_2}Y_{X_3/C}Y_{D/X_3}Y_{X_4/D}\frac{\mu_1}{\mu_4} \tag{35}$$

and that therefore the following relations holds for the product of the yields along the cycle,

$$Y_{A/X_4}Y_{X_1/A}Y_{B/X_1}Y_{X_2/B}Y_{C/X_2}Y_{X_3/C}Y_{D/X_3}Y_{X_4/D} = 1 \tag{36}$$

To understand how it arose, consider the implication of this equation that (upon cancellation of all growth rates and multiplication of the denominator and numerator by the product of the biomasses)

$$\frac{q_A^4 X_4 q_B^1 X_1 q_C^2 X_2 q_D^3 X_3}{q_A^1 X_1 q_B^2 X_2 q_C^3 X_3 q_D^4 X_4} = 1 \tag{37}$$

, which is correct because this equals $J_1J_2J_3J_4/(J_1J_2J_3J_4) = 1$. This equation is reminiscent of the detailed balance equations of that exist for cycles in chemical reaction networks which state that in thermodynamic equilibrium the net free energy change is zero.
